# An epigenetic clock analysis of race/ethnicity, sex, and coronary heart disease

**DOI:** 10.1186/s13059-016-1030-0

**Published:** 2016-08-11

**Authors:** Steve Horvath, Michael Gurven, Morgan E. Levine, Benjamin C. Trumble, Hillard Kaplan, Hooman Allayee, Beate R. Ritz, Brian Chen, Ake T. Lu, Tammy M. Rickabaugh, Beth D. Jamieson, Dianjianyi Sun, Shengxu Li, Wei Chen, Lluis Quintana-Murci, Maud Fagny, Michael S. Kobor, Philip S. Tsao, Alexander P. Reiner, Kerstin L. Edlefsen, Devin Absher, Themistocles L. Assimes

**Affiliations:** 1 Human Genetics, David Geffen School of Medicine, University of California Los Angeles, Los Angeles, CA 90095 USA; 2 Biostatistics, School of Public Health, University of California Los Angeles, Los Angeles, CA 90095 USA; 3 Department of Anthropology, University of California Santa Barbara, Santa Barbara, CA 93106 USA; 4 Department of Anthropology, University of New Mexico, Albuquerque, NM 87131 USA; 5 Department of Preventive Medicine and Institute for Genetic Medicine, Keck School of Medicine, University of Southern California, Los Angeles, CA 90089 USA; 6 Department of Epidemiology, Fielding School of Public Health, University of California Los Angeles, Los Angeles, CA 90095 USA; 7 Longitudinal Studies Section, Translational Gerontology Branch, National Institute on Aging, National Institutes of Health, Baltimore, MD 21224 USA; 8 Department of Medicine, Division of Hematology/Oncology, AIDS Institute, University of California Los Angeles, Los Angeles, CA USA; 9 Department of Epidemiology, Tulane University, New Orleans, LA 70112 USA; 10 Unit of Human Evolutionary Genetics, Centre National de la Recherche Scientifique, URA3012, URA3012 Institut Pasteur, Paris, 75015 France; 11 Department of Biostatistics, Harvard TH Chan School of Public Health and Department of Computational Biology and Biostatistics, Dana-Farber Cancer Institute, Boston, MA 02115 USA; 12 Centre for Molecular Medicine and Therapeutics, Child and Family Research Institute and Department of Medical Genetics, University of British Columbia, Vancouver, BC V5Z 4H4 Canada; 13 Department of Medicine, Stanford University School of Medicine, Stanford, CA 94305 USA; 14 VA Palo Alto Health Care System, Palo Alto, CA USA; 15 Department of Epidemiology, Fred Hutchinson Cancer Research Center, University of Washington, Seattle, WA 98109 USA; 16 Department of Laboratory Medicine, University of Washington, Seattle, WA 98195 USA; 17 HudsonAlpha Institute for Biotechnology, Huntsville, AL 35806 USA

**Keywords:** DNA methylation, Epigenetic clock, Race, Gender, Aging, Coronary heart disease, Hispanic paradox, Black/white mortality cross-over

## Abstract

**Background:**

Epigenetic biomarkers of aging (the “epigenetic clock”) have the potential to address puzzling findings surrounding mortality rates and incidence of cardio-metabolic disease such as: (1) women consistently exhibiting lower mortality than men despite having higher levels of morbidity; (2) racial/ethnic groups having different mortality rates even after adjusting for socioeconomic differences; (3) the black/white mortality cross-over effect in late adulthood; and (4) Hispanics in the United States having a longer life expectancy than Caucasians despite having a higher burden of traditional cardio-metabolic risk factors.

**Results:**

We analyzed blood, saliva, and brain samples from seven different racial/ethnic groups. We assessed the intrinsic epigenetic age acceleration of blood (independent of blood cell counts) and the extrinsic epigenetic aging rates of blood (dependent on blood cell counts and tracks the age of the immune system). In blood, Hispanics and Tsimane Amerindians have lower intrinsic but higher extrinsic epigenetic aging rates than Caucasians. African-Americans have lower extrinsic epigenetic aging rates than Caucasians and Hispanics but no differences were found for the intrinsic measure. Men have higher epigenetic aging rates than women in blood, saliva, and brain tissue.

**Conclusions:**

Epigenetic aging rates are significantly associated with sex, race/ethnicity, and to a lesser extent with CHD risk factors, but not with incident CHD outcomes. These results may help elucidate lower than expected mortality rates observed in Hispanics, older African-Americans, and women.

**Electronic supplementary material:**

The online version of this article (doi:10.1186/s13059-016-1030-0) contains supplementary material, which is available to authorized users.

## Background

Many demographic and epidemiological studies explore the effects of chronological age, race/ethnicity, and sex on mortality rates and susceptibility to chronic disease [1–5], but it remains an open research question whether race/ethnicity and sex affect molecular markers of aging directly. To what extent clinical biomarkers of inflammation, dyslipidemia, and immune senescence relate to cellular markers of aging also remains an open question. One major challenge is the lack of agreement on how to define and measure biological aging rates [6]. Many biomarkers of aging have been proposed ranging from clinical markers (such as whole-body functional evaluations and gait speed) to molecular markers such as telomere length [7, 8]. Available biomarkers capture only particular aspects of aging. For example, African Americans have been shown to have longer telomere lengths than Caucasians [9], despite significantly higher levels of inflammation, lower average life expectancies, and higher disease incidence. To date, no studies have employed epigenetic measures to estimate and compare molecular aging rates among gender or racial/ethnic groups.

Measures incorporating DNA methylation levels have recently given rise to a new class of biomarkers that appear informative of aging given that age has a profound effect on DNA methylation levels in most human tissues and cell types [10–18]. Several recent studies have measured the epigenetic age of tissue samples by combining the DNA methylation levels of multiple dinucleotide markers, known as Cytosine phosphate Guanines or CpGs [19–21]. We recently developed the epigenetic clock (based on 353 CpGs) to measure the age, known as “DNA methylation age” or “epigenetic age,” of assorted human cell types (CD4+ T cells or neurons), tissues, and organs—including blood, brain, breast, kidney, liver, lung [20], and even prenatal brain samples [22]. The epigenetic clock is an attractive biomarker of aging because it applies to most human tissues and its accurate measurement of chronological age is unprecedented.

The following evidence shows that the epigenetic clock captures aspects of biological age. First, the epigenetic age of blood has been found to be predictive of all-cause mortality even after adjusting for chronological age and a variety of known risk factors [23–25]. Second, the blood of the offspring of Italian semi-supercentenarians (i.e. participants who reached an age of at least 105 years) has a lower epigenetic age than that of age-matched controls [26]. Third, the epigenetic age of blood relates to frailty [27] and cognitive/physical fitness in the elderly [28]. The utility of the epigenetic clock method has been demonstrated in applications surrounding obesity [29], Down’s syndrome [30], HIV infection [31], Parkinson’s disease [32], Alzheimer’s disease-related neuropathologies [33], lung cancer [34], and lifetime stress [35]. Here, we apply the epigenetic clock to explore relationships between epigenetic age and race/ethnicity, sex, risk factors of coronary heart disease (CHD), and the CHD outcome itself.

## Results

### Blood datasets and racial/ethnic groups

An overview of our DNA methylation datasets can be found in Table 1. We analyze multiple sources of DNA: mostly blood, saliva, and lymphoblastoid cell lines. In addition, brain datasets were used to compare men and women (Table 2). We considered the following racial/ethnic groups (Table 1): 1387 African Ancestry (African Americans and two groups from Central Africa), 2932 Caucasian (non-Hispanic whites), 657 Hispanic, 127 East Asians (mainly Han Chinese), and 59 Tsimane Amerindians.

**Table 1:** Overview of the DNA methylation datasets. The rows correspond to the datasets used in this article. Columns report the tissue source, DNA methylation platform, number of participants, access information, and citation and a reference to the use in this text

Tissue source	Array	Participants (n)	Women (n)	African Ancestry, Caucasian, Hispanic, Tsimane, East Asian (n)	Mean age (years) (range)	Available	Citation	Figure
1. Women’s Health Initiative (blood)	450	1462	1462	676, 353, 433, 0, 0	63 (50–80)	dbGAP, NHLBI	Current article	1
2. Bogalusa (blood)	450	969	547	288, 681, 0, 0, 0	43 (29–51)	dbGAP, NHLBI	Current article	1
3. PEG (blood)	450	335	138	0, 289, 46, 0, 0	70 (36–91)	GSE72775	Current article	1
4. Saliva from PEG	450	259	113	0, 166, 93, 0, 0	69 (36–88)	GSE78874	Current article	1
5. Older Tsimane and others	450	310	150	0, 235, 38, 37, 0	66 (35–92)	GSE72773	Current article	3
6. Younger Tsimane and Caucasians	450	46	31	0, 24, 0, 22, 0	15 (2–35)	GSE72777	Current article	3
7. East Asians vs. Caucasians (PSP samples removed)	450	312	132	0, 279, 0, 0, 33	68 (34–93)	GSE53740	Li, 2014 [73]	3
8. African populations	450	256	50	256, 0, 0, 0, 0	40 (16–90)	EGAS00001001066	Fagny, 2015 [42]	4
9. Cord blood	27	216	110	92, 70, 0, 0, 0	0 (0–0)	GSE27317	Adkins, 2011 [44]	
10. Male saliva	27	91	0	0, 59, 32, 0, 0	29 (21–55)	GSE34035	Liu, 2010 [74]	
11. Female saliva	27	42	42	0, 27, 15, 0, 0	27 (21–55)	GSE34035	Liu, 2010 [74]	
12. Lymphoblastoid cell lines	450	237	154	75, 68, 0, 0, 94	34 (5–73)	GSE36369	Heyn, 2013 [88]	Additional file 1

**Table 2:** Description of brain datasets for evaluating the effect of gender. Additional details can be found in “Methods”

Data	Participants (n)	Men (%)	Age mean ± SE [min, max]	Brain region	Brain tissue samples (n)
Study 1	117	41 %	84.0 ± 9.8 [40, 105]	CRBLM	112
				EC	108
				PFCTX	114
				STG	117
Study 2	142	68 %	48.0 ± 23.2 [16, 96]	CRBLM	112
				FCTX	133
				PONS	125
				TCTX	127
Study 3	147	63 %	44.3 ± 9.6 [19, 68]	CRLM	147
Study 4	37	62 %	64.4 ± 17.4 [25, 96]	CRBLM	36
				PFCTX	36
Study 5	209	66 %	52.3 ± 29.8 [1, 102]	CRBLM	201
				FCTX	201
Study 6	718	37 %	88.5 ± 6.6 [66, 108]	DLPFC	718

*CRBLM* cerebellum, *DLPFC* dorsolateral prefrontal cortex, *EC* entorhinal cortex, *FCTX* frontal cortex, *PFCTX* prefrontal cortex, *PONS* pons, *STG* superior temporal gyrus, *TCTX* temporal cortex

### Accuracy of the epigenetic clock

**Fig. 1:**
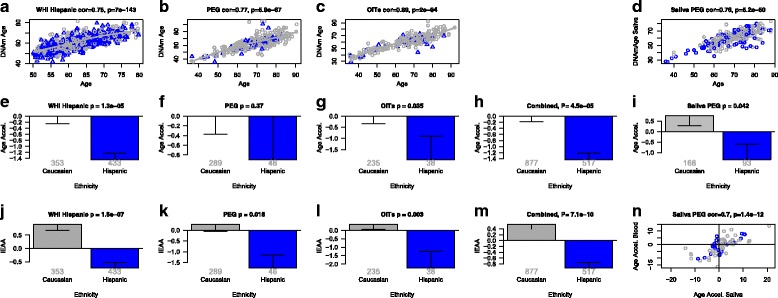
Intrinsic epigenetic age acceleration in Caucasians and Hispanics. **a**-**d** DNA methylation age (*y-axis*) versus chronological age (*x-axis*) in (a) Women’s Health Initiative, (b) blood data from PEG, (c) dataset 5, (d) saliva data from PEG. *Dots* corresponds to participants and are colored by ethnic group (*gray* = Caucasian, *blue* = Hispanic). The *gray line* depicts a spline regression line through Caucasians. We define two measures of age acceleration based on DNAm age. **e**-**g** The *bar plots* relate the universal measure of epigenetic age acceleration to race/ethnicity, which is defined as residual to the spline regression line through Caucasians, i.e. the vertical distance of a point from the line. By definition, the mean age acceleration in Caucasians is zero. **h**, **m** Results after combining the three blood datasets using Stouffer’s meta-analysis method. **i** Age acceleration residual versus ethnicity in the saliva data from PEG. **j**-**m** The *y-axis* reports the mean value of IEAA, which is defined as residual from a multivariate regression model that regresses DNAm age on age and several measures of blood cell counts. Each *bar plot* reports 1 standard error and the *p* value from a group comparison test (ANOVA). **n** Age acceleration in blood versus age acceleration in saliva for the subset of PEG participants for whom both data were available

**Fig. 2:**
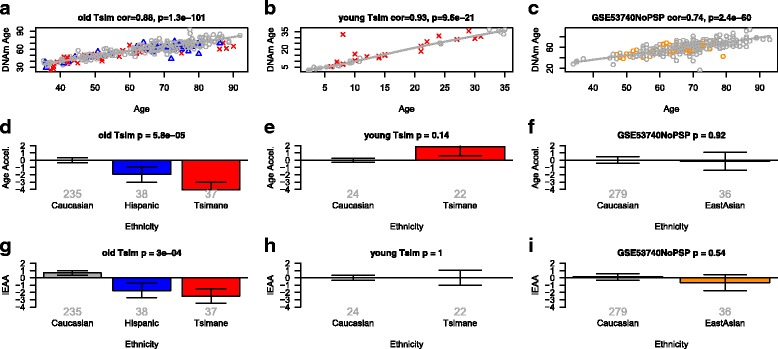
Intrinsic epigenetic age acceleration in Tsimane, Hispanics, East Asians, and Caucasians. **a**-**c** DNA methylation age (*y-axis*) versus chronological age (*x-axis*) in (a) dataset 5, (b) dataset 6, (c) dataset 7. *Dots* corresponds to participants and are colored by race/ethnicity (*green* = African American, *gray* = Caucasian, *blue* = Hispanic, *red* = Tsimane, *orange* = East Asians). The *gray line* depicts a spline regression line through Caucasians. We define two measures of age acceleration based on DNAm age. **d**-**f** The *bar plots* relate the universal measure of epigenetic age acceleration to race/ethnicity, which is defined as residual to the spline regression line through Caucasians, i.e. the vertical distance of a point from the line. **g**-**i** The *y-axis* reports the mean value of IEAA, which is defined as residual from a multivariate regression model that regresses DNAm age on age and several measures of blood cell counts. Each *bar plot* reports 1 standard error and the *p* value from a group comparison test (ANOVA)

**Fig. 3:**
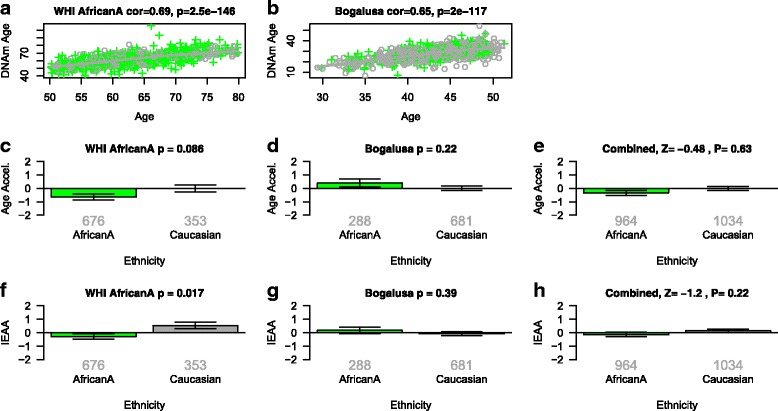
Intrinsic epigenetic age acceleration versus African or European Ancestry. **a**-**c** DNA methylation age (*y-axis*) versus chronological age (*x-axis*) in (a) Women’s Health Initiative, (b) Bogalusa study. *Dots* corresponds to participants and are colored by race/ethnicity (*green* = African Ancestry, *gray* = Caucasian). The *gray line* depicts a spline regression line through Caucasians. We define two measures of age acceleration based on DNAm age. **c**, **d** The *bar plots* relate the universal measure of epigenetic age acceleration to race/ethnicity, which is defined as residual to the spline regression line through Caucasians. **e**, **h** Results after combining the two blood datasets using Stouffer’s meta-analysis method. **f**, **g** The *y-axis* reports the mean value of IEAA, which is defined as residual from a multivariate regression model that regresses DNAm age on age and several measures of blood cell counts. Each bar plot reports 1 standard error and the *p* value from a group comparison test (ANOVA)

**Fig. 4:**
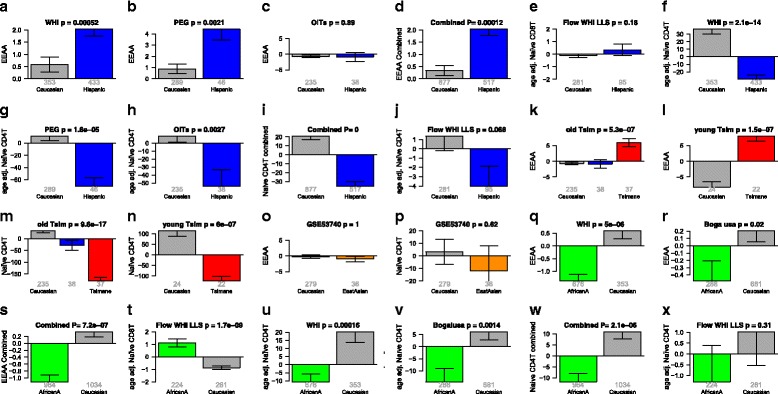
Extrinsic epigenetic age acceleration and blood cell counts across groups. EEAA versus race/ethnicity in (**a**, **q**) Women’s Health Initiative, (**b**) blood data from PEG, (**c**, **k**) dataset 5, (**l**) dataset 6, (**o**) dataset 7, (**r**) Bogalusa study. Flow cytometric, age adjusted estimates (**e**, **t**) naïve CD8+ T and (**j**, **x**) naïve CD4+ T cell counts in the WHI LLS. Age adjusted estimates of naïve CD4 + T cells based on DNA methylation data from (**f**, **u**) Women’s Health Initiative, (**g**) blood data from PEG, (**h**, **m**) dataset 5, (**n**) dataset 6, (**p**) dataset 7, (**v**) Bogalusa study. (**d**, **i**, **s**, **w**) Meta-analysis across the respective datasets based on Stouffer’s method

**Fig. 5:**
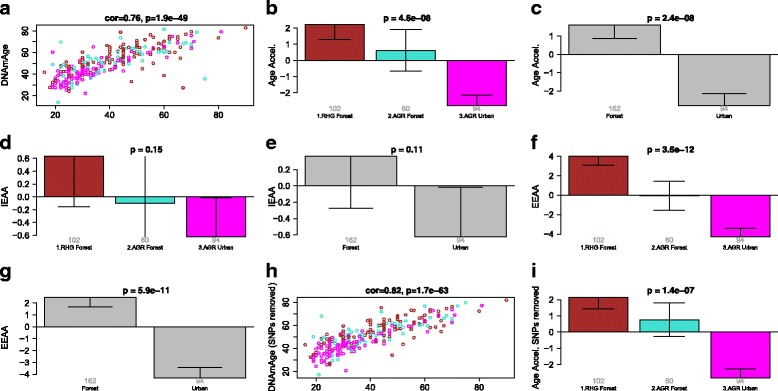
Analysis of African rainforest hunter-gatherers and farmers. **a** DNAm age versus age using 256 blood samples from [42]. The *points* are colored as follows: *magenta* = AGR (urban setting), *turquoise* = AGR (forest), *brown* = RHG (forest). Group status versus (**b**) universal age acceleration, (**d**) intrinsic age acceleration, (**f**) extrinsic age acceleration. Habitat versus (**c**) universal age acceleration, (**e**) intrinsic age acceleration, (**g**) extrinsic age acceleration. (**h**, **i**) are analogous to (**a**, **b**) but the *y-axis* is based on a DNAm age estimate that excluded CpG that were located near SNPs. In this robustness analysis, we removed CpG probes containing genetic variants at a frequency higher than 1 % in the populations studied

DNAm age, also referred to as epigenetic age, was calculated in human samples profiled with the Illumina Infinium 450 K platform using a previously described method [20]. As expected, we found DNAm age to have a strong linear relationship with chronological age in blood and saliva (correlations in the range of 0.65–0.93, Figs. 1, 2, 3, 4, and 5) and in lymphoblastoid cell lines (r = 0.59; Additional file 1). Based on a spline regression line, we defined a “universal” measure of epigenetic age acceleration, denoted “Age Accel.” in our figures, as the difference between the observed DNAm age value and the value predicted by a spline regression model in Caucasians. The term “universal” refers to the fact that this measure can be defined in a vast majority of tissues and cell types with the notable exception of sperm [20]. A positive value of the universal age acceleration measure indicates that DNA methylation age is higher than that predicted from the regression model for Caucasian participants of the same age. Our intrinsic and extrinsic age acceleration measures (see “Methods”) only apply to blood data. A measure of intrinsic epigenetic age acceleration (IEAA) measures cell-intrinsic epigenetic aging effects that are not confounded by extra-cellular differences in blood cell counts. The measure of IEAA is an incomplete measure of the age-related functional decline of the immune system because it does not track age-related changes in blood cell composition, such as the decrease of naïve CD8+ T cells and the increase in memory or exhausted CD8+ T cells [36–38]. The measure of extrinsic epigenetic age acceleration (EEAA) only applies to whole blood and aims to measure epigenetic aging in immune-related components. It keeps track of both intrinsic epigenetic changes and age-related changes in blood cell composition (see “Methods”). The estimated blood cell counts, which are used in these measures, correlate strongly with corresponding flow cytometric measurements from the MACS study (Additional file 2): r = 0.63 for CD8 + T cells, r = 0.77 for CD4+ T, r = 0.67 B cell, r = 0.68 naïve CD8+ T cell, r = 0.86 for naïve CD4+ T, and r = 0.49 for exhausted CD8+ T cells.

### Hispanics have a lower intrinsic aging rate than Caucasians

We find that Hispanics have a consistently lower IEAA compared to Caucasians (*p* = 7.1 × 10^–10^, Fig. 1m). An important question is whether the observed differences in blood can also be observed in other tissues. Using a novel saliva dataset (dataset 4, saliva from PEG) we find that Hispanics have a lower epigenetic aging rate than Caucasians (*p* = 0.042, Fig. 1i). The fact that our findings in blood can also be validated in saliva is consistent with the strong correlation between epigenetic age acceleration measures of the two sources of DNA (r = 0.70, *p* = 1.4 × 10^–12^, Fig. 1n). The lower value of IEAA in Hispanics unlikely reflects country of birth or of residence (at age 35 years) given the robust findings across samples and our detailed analysis in the WHI, where we find that Hispanics born outside US, but living in the US, have a higher IEAA than Hispanics born and raised in the US (*p* = 0.025, Additional file 3B).

### CHD risk factors bear little or no relationship with IEAA

We related our measures of age acceleration to risk factors related to CHD since the latter are significant predictors of mortality. In postmenopausal women from the Women’s Health Initiative (WHI), we found no evidence that IEAA is associated with disparities in education, high density lipoprotein (HDL) or low density lipoprotein (LDL) cholesterol, insulin, glucose, C-reactive protein (CRP), creatinine, alcohol consumption, smoking, diabetes status, or hypertension (see Table 3).

**Table 3:** Multivariate model that regresses epigenetic age acceleration on participant characteristics in the WHI. Coefficients and *p* values from regressing measures of intrinsic and extrinsic epigenetic age acceleration on participant characteristics from dataset 1

Multivariate linear regression		Intrinsic EAA		Extrinsic EAA	
		Estimate (SE)	*p*	Estimate (SE)	*p*
Race/ethnicity	Hispanic vs. African American	–0.94 (0.35)	0.007	3.363 (0.439)	<10^–15^
	White vs. African American	0.71 (0.295)	0.016	1.94 (0.37)	1.6 × 10^–7^
HDL-cholesterol		0.006 (0.01)	0.558	–0.003 (0.013)	0.799
Triglyceride		0.003 (0.002)	0.059	0.004 (0.002)	0.04
Insulin		0 (0.001)	0.664	0.001 (0.001)	0.337
Glucose		0.003 (0.004)	0.486	0.007 (0.005)	0.112
CRP		0.023 (0.018)	0.215	0.052 (0.023)	0.023
Creatinine		0.703 (0.594)	0.237	1.985 (0.745)	0.008
BMI		0.035 (0.021)	0.103	0.045 (0.027)	0.093
Education	High school (HS) vs. no HS	0.357 (0.426)	0.403	–0.784 (0.534)	0.142
	Some college vs. no HS	0.469 (0.381)	0.219	–1.171 (0.478)	0.014
	College vs. no HS	0.486 (0.519)	0.349	–2.253 (0.65)	0.001
	Grad school vs. no HS	0.36 (0.424)	0.396	–1.648 (0.531)	0.002
Alcohol	Past drinker vs. Never	1.668 (1.1)	0.13	–0.598 (1.379)	0.665
	Light drinker vs. Never	–0.101 (0.536)	0.85	–0.751 (0.672)	0.264
	Moderate vs. Never	–0.416 (0.748)	0.578	–0.401 (0.937)	0.669
	Heavy vs. Never	–0.354 (0.88)	0.687	–0.833 (1.103)	0.45
Smoking	Former vs. Current	–0.573 (1.039)	0.581	–0.104 (1.302)	0.936
	Never vs. Current	–0.376 (1.039)	0.718	–0.122 (1.303)	0.925
Diabetes		0.216 (0.43)	0.616	–0.061 (0.539)	0.909
Hypertension		0.364 (0.241)	0.131	0.262 (0.302)	0.386
R-squared		0.029		0.069	

### Tsimane have a lower intrinsic aging rate than Caucasians

The Tsimane are an indigenous population (~15,000 inhabitants) of forager-horticulturalists who reside in the remote lowlands of Bolivia. They reside mostly in open-air thatch huts, and actively fish, hunt, and cultivate plantains, rice, and manioc through slash-and-burn horticulture [39]. Tsimane provide a unique contribution to aging researchers and epidemiologists because they experience high rates of inflammation due to repeated bacterial, viral, and parasitic infections, yet show minimal risk factors for heart disease or type 2 diabetes as they age; they have minimal hypertension and obesity, low LDL cholesterol and no evidence of peripheral arterial disease [39–41]. Since Hispanics share genetic ancestry with peoples indigenous to the Americas, we hypothesized that a slower intrinsic aging rate might also be observable by analyzing Tsimane blood samples [39]. Among participants who are older than 35 years, Tsimane have the lowest intrinsic age acceleration (Fig. 2d, g). While Tsimane have a significantly lower IEAA than Caucasians after the age of 35 years (*p* = 0.0061), no significant difference could be observed in younger participants (Fig. 2e, h). In this analysis, the threshold of 35 years was chosen so that a sufficient number of young participants would be included in dataset 6. We found no significant difference in IEAA between older Hispanics and Tsimane, which might reflect the relatively low group sizes of n = 37 Tsimane versus n = 38 Hispanics.

### IEAA is not associated with CHD in the WHI

Based on our findings above showing little or no relationship between IEAA and CVD risk factors at baseline, we hypothesized that IEAA would not predict future onset of CHD. A multivariate logistic regression model shows that IEAA is not significantly associated with an increased risk of incident CHD (Table 4). However, as expected, current smoking, prior history of diabetes, hypertension, high insulin and glucose levels, and lower HDL predicted an increased risk of CHD (Table 4).

**Table 4:** Logistic model that regresses CHD status on epigenetic age acceleration and participant characteristics in the WHI. Coefficients, Wald Z statistics, and corresponding *p* values resulting from regressing CHD status on measures of epigenetic age acceleration and various participant characteristics. The results for the measure of IEAA and EEAA can be found in columns 2 and 3, respectively

Logistic model. Outcome CHD case status		Intrinsic EAA			Extrinsic EAA		
Covariates		Estimate (SE)	Z	*p*	Estimate (SE)	Z	*p*
Epig. Age Accel		–0.017 (0.01)	–1.72	0.085	–0.006 (0.008)	–0.74	0.458
Age		0.027 (0.008)	3.44	0.001	0.028 (0.008)	3.52	4.3 × 10^-4^
Race/ethnicity	Hispanic vs. African American	0.083 (0.152)	0.55	0.584	0.118 (0.153)	0.77	0.443
	White vs. African American	0.141 (0.135)	1.04	0.298	0.135 (0.135)	1.00	0.319
HDL-cholesterol		–0.02 (0.005)	–4.29	1.8 × 10^–5^	–0.02 (0.005)	–4.33	1.5 × 10^-5^
Triglyceride		0.001 (0.001)	1.43	0.153	0.001 (0.001)	1.38	0.169
Insulin		0.002 (0.001)	2.26	0.024	0.002 (0.001)	2.25	0.024
Glucose		0.005 (0.002)	2.64	0.008	0.005 (0.002)	2.64	0.008
CRP		0.013 (0.008)	1.61	0.107	0.013 (0.008)	1.61	0.108
Creatinine		0.518 (0.281)	1.84	0.065	0.515 (0.281)	1.84	0.067
BMI		–0.011 (0.01)	–1.19	0.235	–0.012 (0.01)	–1.22	0.223
Education	High school (HS) vs. no HS	–0.058 (0.183)	-0.32	0.753	–0.067 (0.183)	–0.37	0.715
	Some College vs. no HS	0.008 (0.164)	0.05	0.96	–0.004 (0.165)	–0.03	0.979
	College vs. no HS	–0.198 (0.223)	–0.89	0.373	–0.219 (0.223)	–0.98	0.327
	Grad school vs. no HS	–0.237 (0.183)	–1.29	0.196	–0.251 (0.183)	–1.37	0.171
Alcohol	Past drinker vs. Never	–0.6 (0.514)	–1.17	0.243	–0.641 (0.513)	–1.25	0.212
	Light drinker vs. Never	–0.34 (0.233)	–1.46	0.145	–0.343 (0.233)	–1.47	0.141
	Moderate vs. Never	–0.1 (0.32)	–0.31	0.754	–0.096 (0.32)	–0.30	0.764
	Heavy vs. Never	–0.34 (0.381)	–0.89	0.373	–0.337 (0.381)	–0.88	0.377
Smoking	Former vs. Current	–0.997 (0.467)	–2.13	0.033	–0.989 (0.467)	–2.12	0.034
	Never vs. Current	–1.321 (0.468)	–2.82	0.005	–1.317 (0.468)	–2.81	0.005
Diabetes		0.706 (0.196)	3.61	3.0 × 10^-4^	0.699 (0.196)	3.58	3.4 × 10^-4^
Hypertension		0.565 (0.103)	5.46	4.8 × 10^-8^	0.559 (0.103)	5.41	6.3 × 10^-8^

### Hispanics and Tsimane have a higher EEAA than Caucasians

According to our measure of EEAA, Hispanics have a significantly older extrinsic epigenetic age than Caucasians (meta-analysis *p* = 0.00012, Fig. 4a–d) and fewer naïve CD4+ T cells, based on cytometric data from the WHI LLS, the MACS study, and imputed blood cell counts (Fig. 4f–j, Additional file 2H, I). This pattern of fewer naïve CD4+ T cells is even more pronounced for Tsimane (Fig. 4m, n), who experience repeated acute infections and elevated, often chronic, inflammatory loads.

### Epigenetic age analysis of East Asians

Because ancient Native American populations share common ancestral lineages with East Asians, we examined whether East Asians also differ from Caucasians in terms of epigenetic aging rates. We found no significant difference between Caucasians and East Asians in terms of IEAA (Fig. 2i), EEAA (Fig. 4o), or naïve CD4+ T cells (Fig. 4p). Similarly, we found no difference in lymphoblastoid cell lines (Additional file 1). However, these comparative analyses are limited by the relatively small number of samples and should be repeated in larger datasets.

### Which risk factors for cardiometabolic disease are associated with EEAA?

Our multivariate model analysis in the WHI (Table 3) shows that EEAA tracks better than IEAA with risk factors for cardiometabolic disease; EEAA was positively associated (higher) with: triglyceride levels (multivariate model *p* = 0.04), CRP (*p* = 0.023), and creatinine (*p* = 0.008). EEAA was negatively associated (lower) with higher levels of education in all ethnic groups (*p* from 2.0 × 10^–8^ to 0.05, Additional file 4I–L). For each racial/ethnic group, we find that women who did not finish high school exhibit the highest levels of EEAA (leftmost bar in Additional file 4J–L).

### Epigenetic aging rates of African Americans

In the following, we compare African Americans with European Americans in terms of IEAA and EEAA. Comparisons of African Americans with Caucasians in terms of IEAA yield contradictory findings across datasets that differ in age range: African American women have slightly lower IEAA than Caucasian women in the WHI (*p* = 0.017 Fig. 3f), but no significant difference can be observed for the younger participants of the Bogalusa study (Fig. 3g). Indeed, participants in the WHI (aged between 50 and 80 years) were older than those of the Bogalusa study (aged between 29 and 51 years). This failure to detect a significant racial/ethnic difference in IEAA in younger participants is consistent with our results from the comparison of younger Tsimane and Caucasians (Fig. 2h). A multivariate model analysis based on the Bogalusa study (comprising African Americans and Caucasians) confirms that IEAA does not differ between middle-aged African Americans and Caucasians but IEAA is higher among men (*p* = 0.025) and has a marginally significant association with hypertension (*p* = 0.064, Table 5). When relating individual variables to IEAA, we find significant associations for hypertension (*p* = 0.00035, Additional file 5D–F) but not for type II diabetes status or educational level.

**Table 5:** Multivariate model that regresses epigenetic age acceleration on participant characteristics in the Bogalusa study. Coefficients and *p* values from regressing measures of intrinsic and extrinsic epigenetic age acceleration on participant characteristics from dataset 2

Multivariate linear regression		Intrinsic EAA			Extrinsic EAA		
		Estimate (SE)	Z	*p*	Estimate (SE)	Z	*p*
Race	Caucasian vs. African American	–0.013 (0.316)	–0.04	0.97	0.843 (0.316)	2.67	0.0076
Gender	Female vs. Male	–0.622 (0.278)	–2.24	0.025	–0.718 (0.277)	–2.60	0.0093
Education	Grade 8–9 vs. < Grade 8	1.583 (1.468)	1.08	0.28	2.177 (1.465)	1.49	0.14
	Grade 10–12 vs. < Grade 8	1.285 (1.27)	1.01	0.31	2.267 (1.267)	1.79	0.074
	Vocat/Tech vs. < Grade 8	0.307 (1.299)	0.24	0.81	1.921 (1.295)	1.48	0.14
	College vs. < Grade 8	0.85 (1.281)	0.66	0.51	2.375 (1.277)	1.86	0.062
	Graduate vs. < Grade 8	0.147 (1.336)	0.11	0.91	1.53 (1.332)	1.15	0.25
Diabetes (II)		0.173 (0.485)	0.36	0.72	0.012 (0.483)	0.03	0.98
Hypertension		0.539 (0.291)	1.86	0.064	1.247 (0.29)	4.30	1.7 × 10^-5^
R-squared		0.025			0.043		

Our findings for EEAA are highly consistent across the two studies and age groups: African Americans have lower EEAA than Caucasians in the WHI and in the Bogalusa study (*p* = 7.2 × 10^–7^, Fig. 4q, r, s). Our flow cytometric data from the WHI LLS show that African American women exhibit a higher abundance of naïve CD8+ T cells than Caucasian women (*p* = 1.7 × 10^–9^, Fig. 4t).

In multivariate regression analyses of EEAA, we find that African Americans have indications of a significantly younger immune system age than Caucasians (*p* = 0.0076) after controlling for gender, educational level, diabetes status, and hypertension. In the Bogalusa study, we find three significant predictors of EEAA: race/ethnicity, hypertension, and gender (*p* = 0.0093, Table 5). A marginal analysis in the Bogalusa study identifies a significant association between EEAA and hypertension (*p* = 8.0 × 10^–5^, Additional file 5G–I), type II diabetes status in Caucasians (*p* = 0.0085, Additional file 6H), but not in African Americans (Additional file 6I). Contrary to our findings in the WHI, no significant association can be observed between EEAA and educational level (Additional file 7).

### African rainforest hunter-gatherers and farmers

To evaluate the effect of subsistence ecology and environment on epigenetic aging rates, we analyzed 256 blood samples from two different groups in Central Africa: rainforest hunter-gatherers (RHGs, traditionally known as “pygmies,” sampled from Baka and Batwa populations) and African populations that have adopted an agrarian lifestyle (AGRs, traditionally known as “Bantus,” sampled from the Nzebi, Fang, Bakiga, and Nzime populations) over the last 5000 years [42]. The ancestors of the RHGs and AGRs diverged ~60,000 years ago. These groups have historically occupied separate ecological habitats—the ancestors of RHGs in the equatorial rainforest while those of AGRs in drier, more open space savannahs and grasslands. Many RHG groups still live in the rainforest as mobile bands, whereas AGR populations now occupy primarily rural or urban deforested areas, though some AGR groups have settled in the rainforest over the last millennia.

We considered three groups: (1) RHG (n = 102); (2) AGR living in the forest (n = 60); and (3) AGR living in an urban setting (n = 94). The forest habitat was significantly associated with an increase in *AgeAccel* (*p* = 2.4 × 10^–8^, Fig. 5c) and EEAA (*p* = 5.9 × 10^–11^, Fig. 5g), but no difference was found for IEAA (*p* = 0.11, Fig. 5e). Further, no significant difference could be observed between AGR and RHG when focusing on participants living in the rainforest, suggesting greater importance of environment over genetic differences. These results are not affected by differences in genetic variants between RHG and AGR as can be seen from a robustness analysis where we removed CpG probes containing genetic variants at a frequency higher than 1 % in the populations studied (Fig. 5h, i).

### Sex effects in blood and saliva

We explored whether differences exist between men and women in epigenetic aging rates. According to measures of IEAA, men are older than women in two racial/ethnic groups: African Americans (Additional file 8A, B) and Caucasians (Additional file 9A, B, N, Z).

**Fig. 6:**
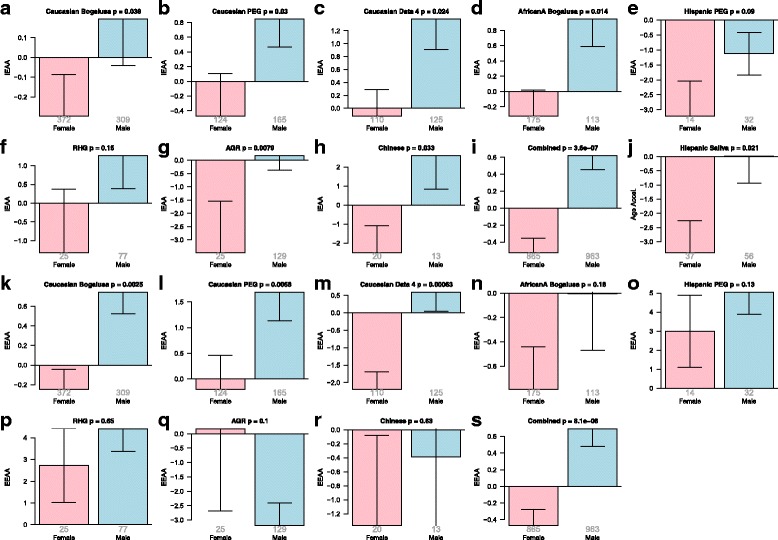
Sex effect on epigenetic age acceleration in blood and saliva. *Panels* of the first two rows (**a**-**j**) and last two rows (**k**-**s**) relate sex to intrinsic and extrinsic epigenetic age acceleration, respectively. Results are reported for blood tissue in all but one panel (**j**). The combined results across all blood studies can be found in panels (**i**) IEAA, (**s**) EEAA. Each *bar plot* reports 1 standard error and a Kruskal–Wallis test

Overall, men have higher IEAA and EEAA than women even when controlling for education, diabetes, and hypertension (Table 5). Using saliva data from PEG, we find that Hispanic men age faster than Hispanic women (*p* = 0.021, Fig. 6j). According to EEAA, Caucasian men are epigenetically older than Caucasian women (Additional file 9C, O, ZA), but we do not observe a significant difference in other groups such as African Americans (Additional file 8C) or central African populations (Fig. 6p, q). The results for EEAA are also consistent with significant sex differences in blood cell counts suggesting more rapid immunosenescence in men. Men have fewer naïve CD4+ T cells than women in three racial/ethnic groups: Caucasians (*p* = 0.0015 in the Bogalusa study, *p* = 0.051 in PEG, *p* = 4.2 × 10^–5^ in dataset 5); Tsimane (*p* = 0.0088 in older Tsimane); and African Americans (*p* = 0.011 in the Bogalusa study).

### Sex effects in brain tissue

**Fig. 7:**
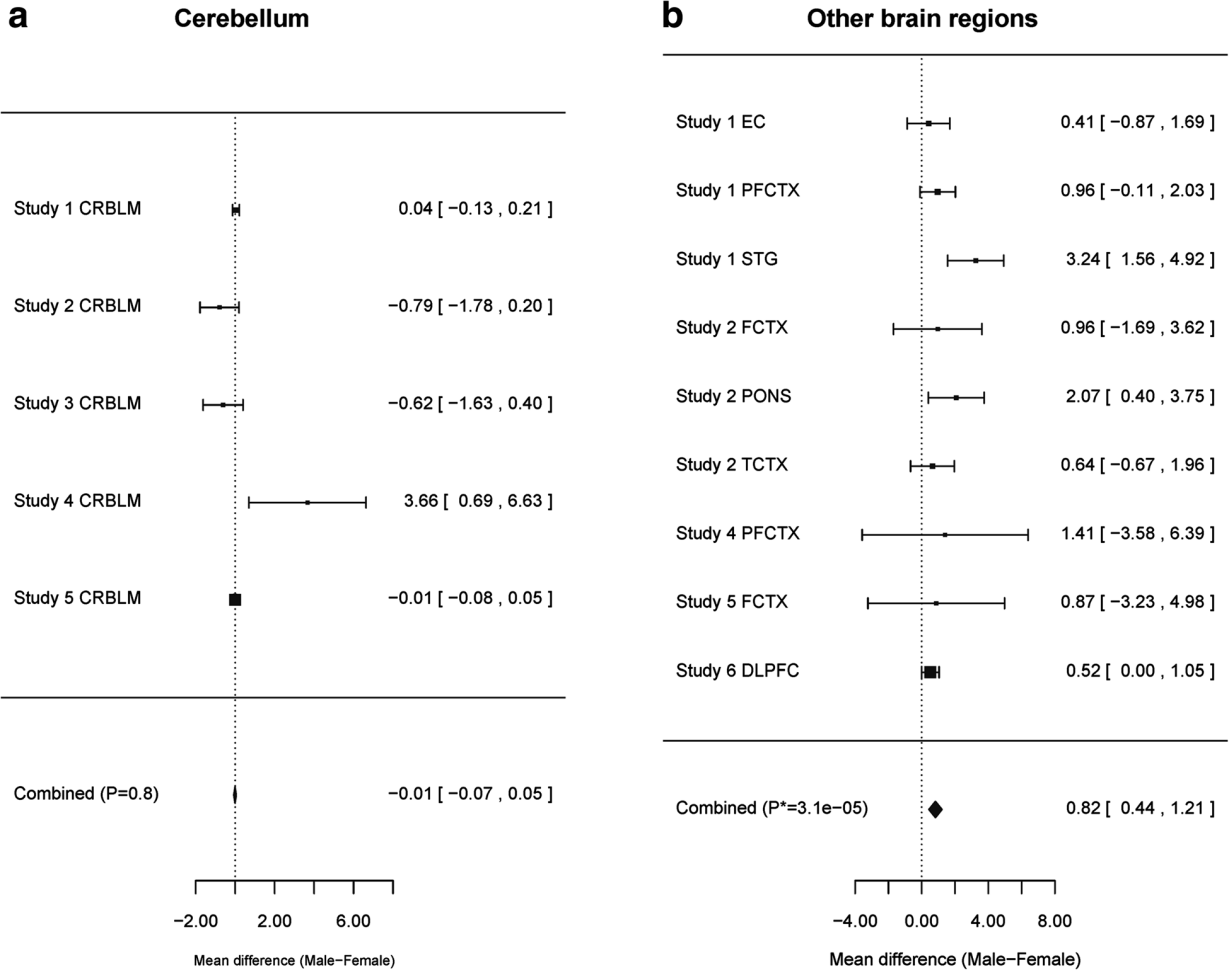
Effect of sex on the epigenetic age of brain tissue. Each *panel* depicts a *forest plot* resulting from the meta-analysis of sex effects. Each *row* in a *forest plot* shows the mean difference in epigenetic age between men and women and a 95 % confidence interval. To combine the coefficient estimates from the respective studies into a single estimate, we applied a fixed-effects model weighted by inverse variance, which is implemented in the *metafor* R package [89]. **a** Gender did not have a significant effect on the epigenetic age of the cerebellum, which is known to age more slowly than other brain regions according to the epigenetic clock [43]. **b** When excluding cerebellar samples from the analysis, we find that male brain regions exhibit a significantly higher age acceleration than female brain regions (mean difference = 0.82, meta-analysis *p* = 3.1 × 10^–5^). The difference remains significant even after adjusting for intra-subject correlations using a linear mixed effects model (mean difference = 0.77, *p* = 0.0034)

We analyzed the effect of sex on the universal measure of age acceleration (Age Accel.) in six independent brain datasets (Table 2 and “Methods”). In total, we analyzed 2287 brain samples from 1370 participants. In our analysis, we distinguished the cerebellum from other brain regions because it is known to age more slowly than other brain regions according to the epigenetic clock [43]. While sex did not have a significant effect on the epigenetic age of the cerebellum (Fig. 7a), we found that other brain regions from men exhibit a significantly higher age acceleration than those from women (Fig. 7b, meta-analysis *p* = 3.1 × 10^–5^).

### Studies of young participants

So far, our results have largely pertained to participants who are middle-aged or older (Table 1, column 6) as we only had access to two datasets involving newborns, infants, children, adolescents, and/or young adults. In dataset 6 (which involved participants between the ages of 2 and 35 years), we did not observe a significant difference epigenetic aging rates between Caucasians and Tsimane. In cord blood samples [44], we found no significant difference in the epigenetic ages of cord blood samples between African American and Caucasian newborns (*p* = 0.23).

### Robustness analysis in the WHI

The epigenetic clock involves 47 CpGs whose broadly defined neighborhood includes a single nucleotide polymorphism (SNP) marker according to the probe annotation file from the Illumina 450 K array. Thus, genetic differences coupled with differences in hybridization efficiency could give rise to spurious differences between different racial/ethnic groups.

We addressed this concern in multiple ways. First, we re-analyzed the WHI data by removing the 47 CpGs (out of 353 epigenetic clock CpGs) from the analysis. The epigenetic clock software imputes the 47 missing CpGs using a constant value (the mean value observed in the original training set). Using the resulting modified epigenetic clock, we validate our findings of racial/ethnic differences in terms of IEAA and EEAA (Additional file 8A–C). However, this type of robustness analysis is limited because the removal of a subset of DNA methylation probes, potentially influenced by proximal genetic variation, is not as good a control as directly having matched genetic data. Second, we used a completely independent epigenetic biomarker based on a published signature of age-related CpGs from Teschendorff et al. [13]. Again, these results corroborate our findings (Additional file 8D, E). Third, we validated our findings using the original blood-based aging measure by Hannum [19] (Additional file 8F, G). Fourth, we highlight that both the Horvath and Hannum age estimators were developed based on training data from mixed populations. The training data underlying the Horvath clock involved four racial/ethnic groups (mainly Caucasians, Hispanics, African Americans, and to a lesser extent East Asians). The Hannum clock was trained on Caucasians and Hispanics. While race/ethnicity can lead to a significant offset between DNAm age and chronological age (which is interpreted as age acceleration), these two variables are highly correlated in all racial/ethnic groups.

## Discussion

Our main findings are that: (1) Hispanics and Tsimane have a lower intrinsic but a higher extrinsic aging rate than Caucasians; (2) African Americans have a lower extrinsic epigenetic aging rate than Caucasians and Hispanics; (3) levels of education are associated with a decreased level of EEAA in each race/ethnic group (Additional file 4); (4) neither intrinsic nor extrinsic aging rates of blood tissue are predictive of incident CHD in the WHI even though EEAA is weakly associated with several cardiometabolic risk factors of CHD (such as hypertension, triglycerides, and CRP); (5) men exhibit higher epigenetic aging rates than women in blood, saliva, and brain samples, and (6) the rain forest habitat is significantly associated with extrinsic age acceleration but not with intrinsic age acceleration in African populations. Although precise understanding of the significance of epigenetic aging measures awaits further elaboration, our principal findings may provide additional context towards resolving several controversial, epidemiological paradoxes, including the Hispanic paradox, black–white mortality cross-over, the Tsimane inflammation paradox, and the sex morbidity–mortality paradox.

### Hispanic paradox

The lower level of IEAA in Hispanics echo the finding that Hispanics in the US have a lower overall risk of mortality than Caucasians despite having a disadvantaged risk profile [45–48]. Our findings stratified by country of birth suggest that the lower intrinsic aging rate of Hispanics does not reflect biases arising through immigration such as a “healthy immigrant effect” (Additional file 3). Our finding regarding higher levels of EEAA in Hispanics parallels the findings that Hispanics have higher levels of metabolic/inflammatory risk profiles [49] and that Hispanics have a lower relative CD4+ T cell percentage than Caucasians [50]. Several articles have explored the question of why the immune system of Hispanics might differ from that of Caucasians [51–53].

### Black–white mortality cross-over

In the US, the black–white mortality cross-over refers to the reported pattern of lower mortality after the age of 85 years among black men and women, compared to whites, despite their higher observed mortality rates at younger ages [54–57]. Although we find no differences in IEAA between African Americans and Caucasians at younger ages, older African American adults from the Bogalusa study had lower IEAA than their Caucasian counterparts. This finding might reflect selective survival of more robust individuals or other aspects of health and systemic risk given its independence from common risk factors for cardiovascular disease and type II diabetes mellitus. Our finding regarding the lower EEAA of African Americans, compared to Caucasians, is consistent with the longer leukocyte telomere lengths of African Americans relative to those of Caucasians [3, 9]. Lastly, our flow cytometric data show that African Americans have a larger number of naïve CD8+ T cells than Caucasians (Fig. 4t).

### Tsimane inflammation paradox

Our results regarding the low intrinsic aging rate in Tsimane may help address another paradox (which we refer to as the Tsimane inflammation paradox), wherein high levels of inflammation and infection, and low HDL levels, are not associated with accelerated cardiovascular aging [39]. The finding that Tsimane have decreased levels of IEAA has parallels to the following clinical/epidemiological observations: even older Tsimane show little evidence of chronic diseases common in high-income countries, like diabetes, atherosclerosis, asthma, and other autoimmune disorders [39]. High levels of physical activity are maintained well into late adulthood [58].

The finding that Tsimane have increased levels of EEAA has parallels to the following observation: a lifetime of diverse pathogen stresses, elevated inflammation and extensive immune activation, seems to lead to more rapid depletion of naïve CD4+ T cells and greater expression of exhausted T cells, i.e. more rapid immunosenescence [39, 40, 59]. Infectious disease and high chronic inflammatory load contribute to the low life expectancy of Tsimane, 43.5 years at birth during the period 1950–1989, and 54.1 years during 1990–2002 [40, 60].

### Sex morbidity–mortality paradox

The sex morbidity–mortality paradox was first described in the 1970s and refers to the observation that women possess a lower age-adjusted mortality rate compared to men despite a higher suffering from a higher burden of co-morbid conditions [61, 62]. Most explanations focus on differences in lifestyle behaviors or healthcare utilization. However, marked sex differences in health and disability remain after controlling for differences in work-related behavior, smoking, obesity, and other behaviors [63]. Whereas other explanations attest to sex differences in a variety of biomarkers, our epigenetic aging markers show robust and consistent male-biased vulnerability in multiple tissues (blood, brain, and saliva) in all racial groups. Similar sex differences in blood-based epigenetic aging rates have also been reported in minors and teenagers [64].

### Strengths and limitations

Our study has several strengths including the analysis of 18 DNA methylation datasets (Tables 1 and 2), large sample sizes (almost 6000 samples), multiple tissues (blood, saliva, brain), access to unique populations (Tsimane Amerindians; rainforest hunter-gatherers and farmers), two flow cytometric studies, and robust epigenetic biomarkers of aging. Our analysis of race/ethnicity also spanned seven different racial/ethnic groups (African American, Caucasian, Hispanic, Tsimane, East Asian, RHGs, and AGRs from Central Africa). Another strength is that our analysis of race/ethnicity involved two sources of DNA: blood and saliva. Limitations include the use of some datasets that are cross-sectional as opposed to longitudinal datasets and the fact that both IEAA and EEAA rely on imputed blood cell counts based on DNA methylation levels. Fortunately, the imputed blood cell counts are quite accurate (Additional file 2). Our results reported here concerning ethnic/racial differences in blood cell counts are supported both by our two flow cytometric datasets and by the literature. However, these measured data are not fully reflective of the breakdown of blood cell types, representing only T and B cells.

## Conclusion

Our exploratory study demonstrates that epigenetic aging rates differ between different racial/ethnic groups and between men and women. Further, intrinsic epigenetic aging rates tend to have insignificant associations with well-studied risk factors of CHD whereas extrinsic aging rates tend to have significant (but weak) associations with several pro-inflammatory risk factors. While racial/ethnic differences have previously been observed in DNA methylation levels [44], we are the first to directly compare epigenetic aging rates across different racial/ethnic groups. Our derived intrinsic and extrinsic epigenetic aging rates in blood offer an independent glimpse into biological aging that incorporates genetics and the environment and provides potential insight into a number of epidemiological paradoxes. The application of genome-wide DNAm-based epigenetic analysis to understand race/ethnic and sex disparities in biological aging is novel and offers an important perspective that complements existing approaches based on other biomarkers. Future studies will need to confirm our findings with longitudinal designs and to extend the epigenetic age analysis to other tissues and organs.

## Methods

We differentiate groups according to “race/ethnicity,” mindful about existing controversies over rigid racial definitions. Our use of these terms reflects self-identified group membership based on macro-categories commonly employed in censuses, human genetics, demography, and epidemiology. The term race/ethnicity thus combines elements of genetic ancestry, population history, and culture.

### DNA methylation age and epigenetic clock

All of the described epigenetic measures of aging and age acceleration are implemented in our freely available software. The epigenetic clock is defined as a prediction method of age based on the DNAm levels of 353 CpGs. Predicted age, referred to as DNAm age, correlates with chronological age in sorted cell types (CD4+ T cells, monocytes, B cells, glial cells, neurons), tissues, and organs, including: whole blood, brain, breast, kidney, liver, lung, saliva [20]. Mathematical details and software tutorials for the epigenetic clock can be found in the Additional files of [20]. An online age calculator can be found at our webpage (https://dnamage.genetics.ucla.edu).

### Intrinsic versus extrinsic measures of epigenetic age acceleration in blood

Empirical studies show that DNAm has a relatively weak correlation with various measures of white blood cell counts [31], which probably reflects the fact that dozens of different tissue and blood cell types were used to define DNAm age. However, we find it useful to explicitly define another measure of age acceleration that is completely independent of blood cell counts as described in the following. We distinguish intrinsic from extrinsic measures of epigenetic age acceleration in whole blood according to their relationship with blood cell counts. A measure of intrinsic epigenetic age acceleration (IEAA) measures “pure” epigenetic aging effects that are not confounded by differences in blood cell counts. Our measure of IEAA is defined as the residual resulting from a multivariate regression model of DNAm age on chronological age and various blood immune cell counts (naïve CD8+ T cells, exhausted CD8+ T cells, plasma B cells, CD4+ T cells, natural killer cells, monocytes, and granulocytes). The measure of IEAA is an incomplete measure of the age-related functional decline of the immune system because it does not track age-related changes in blood cell composition, such as the decrease of naïve CD8+ T cells and the increase in memory or exhausted CD8+ T cells [36–38].

We defined a measure of EEAA that only applies to whole blood and aims to measure epigenetic aging in immune-related components in two steps. First, we formed a weighted average of the epigenetic age measure from Hannum et al. [19] and three estimated measures of blood cells for cell types that are known to change with age: naïve (CD45RA + CCR7+) cytotoxic T cells; exhausted (CD28-CD45RA-) cytotoxic T cells; and plasma B cells using the approach by Klemera Doubal [65]. Second, we defined the measure of EEAA as the residual resulting from a univariate model that regressed the weighted average on chronological age. By definition, our measure of EEAA has a positive correlation with the amount of exhausted CD8+ T cells and plasmablast cells and a negative correlation with the amount of naïve CD8+ T cells. Blood cell counts were estimated based on DNA methylation data. EEAA tracks both age-related changes in blood cell composition and intrinsic epigenetic changes. In most blood datasets, EEAA has a moderate correlation (r = 0.5) with IEAA. We note that, by definition, none of our three measures of epigenetic age acceleration are associated with the chronological age of the participant at the time of blood draw.

### Relationship to mortality prediction

Although the epigenetic clock method was only published in 2013, there is already a rich body of literature that shows that it relates to biological age. Using four human cohort studies, we previously demonstrated that both the Horvath and Hannum epigenetic clocks are predictive of all-cause mortality [23]. Published results in Marioni et al. [23] show that DNAm age adjusted for blood cell counts (i.e. IEAA) is prognostic of mortality in four cohort studies. We recently expanded our original analysis by analyzing 13 different cohorts (including three racial/ethnic groups) and by evaluating the prognostic utility of both IEAA and EEAA. All considered measures of epigenetic age acceleration were predictive of age at death in univariate Cox models (p_AgeAccel_ = 1.9 × 10^–11^, p_IEAA_ = 8.2 × 10^–9^, p_EEAA_ = 7.5 × 10^–43^) and multivariate Cox models adjusting for risk factors and pre-existing disease status (p_AgeAccel_ = 5.4 × 10^–5^, p_IEAA_ = 5.0 × 10^–4^, p_EEAA_ = 3.4 × 10^–19^) where the latter adjusted for chronological age, body mass index, education, alcohol, smoking pack years, recreational physical activity, and prior history of disease (diabetes, cancer, hypertension). These results will be published elsewhere. Further, the offspring of centenarians age more slowly than age matched controls according to *Age Accel* and IEAA [26] which strongly suggests that these measures relate to heritable components of biological age. Two independent research groups have shown that epigenetic age acceleration predicts mortality [24, 25].

### Description of the blood datasets listed in Table 1

All data presented in this article have been made publicly available as indicated in the column “Available” of Table 1.

#### Dataset 1: Women’s Health Initiative (WHI)

Participants included a subsample of participants of the WHI study, a national study that began in 1993 which enrolled postmenopausal women between the ages of 50 and 79 years into either one of two three randomized clinical trials [66]. None of these women had CHD at baseline but about half of these women had developed CHD by 2010. Women were selected from one of two WHI large subcohorts that had previously undergone genome-wide genotyping as well as profiling for seven cardiovascular disease related biomarkers including total cholesterol, HDL, LDL, triglycerides, CRP, creatinine, insulin, and glucose through two core WHI ancillary studies [67]. The first cohort is the WHI SNP Health Association Resource (SHARe) cohort of minorities that includes >8000 African American women and >3500 Hispanic women. These women were genotyped through WHI core study M5-SHARe (www.whi.org/researchers/data/WHIStudies/StudySites/M5) and underwent biomarker profile through WHI Core study W54-SHARe (…data/WHIStudies/StudySites/W54). The second cohort consists of a combination of European Americans from the two Hormonal Therapy trials selected for GWAS and biomarkers in core studies W58 (…/data /WHIStudies/StudySites/W58) and W63 (…/data/WHIStudies/StudySites/W63). From these two cohorts, two sample sets were formed. The first (sample set 1) is a sample set of 637 CHD cases and 631 non-CHD cases as of 30 September 2010. The second sample set (sample set 2) is a non-overlapping sample of 432 cases of CHD and 472 non-cases as of 17 September 2012. The ethnic groups differed in terms of the age distribution in the sense that Caucasian women tended to be older. Therefore, we randomly removed 80 % of the Caucasian women who were older than 65 years when it came to the direct comparisons reported in our figures. This resulted in a total sample size of 1462 women, comprising 673 African Americans, 353 Caucasians, and 433 Hispanics. There was no significant difference in age between the three ethnic groups. However, we kept all of the samples in our analysis of clinical characteristics, such as future CHD status and baseline characteristics such as education, hypertension, diabetes, and smoking, in order to ensure that sufficient sample sizes were available for these analyses. Our results are highly robust with respect to using the smaller or larger versions of the datasets. All results are qualitatively the same for the two versions of the datasets. We acknowledge a potential for selection bias using the above-described sampling scheme in WHI but suspect if such bias is present it is minimal. First, some selection bias is introduced by restricting our methylation profiling at baseline to women with GWAS and biomarker data from baseline as well, given the requirement that these participants must have signed the WHI supplemental consent for broad sharing of genetic data in 2005. However, we believe that selection bias at this stage is minimized by the inclusion of participants who died between the time of the start of the WHI study and the time of supplemental consent in 2005, which resulted in the exclusion of only ~6–8 % of all WHI participants. Nevertheless, participants unable or unwilling to sign consent in 2005 may not represent a random subset of all participants who survived to 2005. Second, some selection bias may also occur if similar gross differences exist in the characteristics of participants who consented to be followed in the two WHI extension studies beginning in 2005 and 2010 compared to non-participants at each stage. We believe these selection biases if present have minimal effects on our effect estimates. Data are available from the page https://www.whi.org/researchers/Stories/June%202015%20WHI%20Investigators'%20Datasets%20Released.aspx, see the link https://www.whi.org/researchers/data/Documents/WHI%20Data%20Preparation%20and%20Use.pdf.

#### Dataset 2: Bogalusa

We analyzed the blood DNA methylation levels of 968 participants (680 Caucasians, 288 African Americans; age range = 28–51.3 years) from the Bogalusa Heart study [68] who were examined in Bogalusa, Louisiana during 2006–2010 for cardiovascular risk factors. All participants in this study gave informed consent at each examination. Study protocols were approved by the Institutional Review Board (IRB reference no. 12-395283) of the Tulane University Health Sciences Center. DNA was extracted from 1106 whole blood samples using the PureLink Pro 96 Genomic DNA Kit (LifeTechnology, CA, USA) following the manufacturer’s instructions. The Infinium HumanMethylation450 BeadChip (Methy450K) was used for whole genome DNA methylation analysis.

All the samples were processed at the Microarray Core Facility, University of Texas Southwestern Medical Center at Dallas, Texas. For DNA methylation analysis, 750 ng genomic DNA from each participant was bisulphite converted using the EZ-96 DNA Methylation Kit (Zymo Research, CA, USA) and the efficiency of the bisulphite conversion was confirmed by built-in controls on the Methy450K array. The methylation profile of each individual was measured by processing 4 μL of bisulphite-converted DNA, at a concentration of 50 ng/μL, on a Methy450K array. The bisulphite-converted DNA was amplified, fragmented, and hybridized to the array. The arrays were scanned on an Illumina HiScan scanner and the raw methylation data were extracted using Illumina’s Genome Studio methylation module. Data cleaning procedures were undertaken using R package “minfi” [69], generating quality control report, finding sample outliers, cell counts estimation, and annotation accessing. The R package wateRmelon [70] was used for β-value normalization and quality control. For correction of systematic technical biases in the 450 K assay, β-value normalization was performed by the “dasen” function, in which type I and type II intensities and methylated and unmethylated intensities will be quantile normalized separately after backgrounds equalization of type I and type II. The R package ChAMP [71] was used for batch effect analysis and correction with “champ.SVD” and “champ.runCombat” functions. The clinical variables and participant characteristics are defined in the captions of the respective Additional files.

The are available from https://biolincc.nhlbi.nih.gov/studies/bhs/.

#### Dataset 3: blood from Hispanics and Caucasians of PEG

The Parkinson’s disease, Environment, and Genes (PEG) case-control study aims to identify environmental risk factors (e.g. neurotoxic pesticide exposures) for Parkinson’s disease.

The PEG study is a large population-based study of Parkinson’s disease of mostly rural and township residents of California’s central valley [72]. Here we only used diseased participants from wave 1 (PEG1). Since all participants of dataset 3 had Parkinson’s disease, disease status could not confound associations with epigenetic aging. Medication status was not associated with epigenetic age acceleration. The data are available from Gene Expression Omnibus.

#### Dataset 4: saliva samples from PEG

This novel dataset comes from the PEG study (described above). Since PD disease status did not relate to epigenetic age acceleration in these data, we ignored it in the analysis. However, our findings are unchanged after incorporating PD status in a multivariate model. About half of the samples overlapped with those of dataset 3, which is why we could correlate epigenetic age acceleration between blood and saliva.

#### Datasets 5 and 6: blood from Tsimane, Hispanics, and Caucasians

Datasets 5 and 6, which were collected and generated in the same way, only differ in terms of the chronological ages. All participants in dataset 5 are older than 35 years while those in dataset 6 are younger or equal to 35 years. The dataset involved three different ethnic groups: Tsimane Amerindians, Hispanics living in the US, and Caucasians living in the US. Fasting whole-blood samples were collected from Tsimane via venipuncture in field villages in the vicinity of San Borja, Bolivia as a part of the annual biomedical data collection for a longitudinal project on aging during 2004–2009 (Tsimane Health and Life History Project). Manual complete blood counts were conducted using a hemocytometer, erythrocyte sedimentation rate was calculated following the Westergren method, and hemoglobin was analyzed with a QBC Autoread Plus Dry Hematology System (Drucker Diagnostics, Port Matilda, PA, USA). Specimens were stored in liquid nitrogen until transfer to the US on dry ice, where they were stored at –80 °C. All participants provided written and informed consent; study protocols and procedures were approved at the individual, village, and Tsimane government level, as well as by the University of California, Santa Barbara and University of New Mexico Institutional Review Boards (IRB Reference numbers 14-0604 and 07-157, respectively). Specimens were shipped on dry ice to the University of Southern California for extraction. The same core facility provided blood samples that were collected at the same time and stored in the same condition as Hispanic participants living in the US. The DNA samples from all participants (Caucasians, Hispanics, Tsimane) were randomized across the Illumina chips to avoid confounding due to chip effects. For our age prediction analysis, we used background corrected beta values resulting from Genome Studio.

Hispanics for datasets 5 + 6: *Participant recruitment*: Participation in the BetaGene study was restricted to Mexican Americans from families of a proband with gestational diabetes mellitus (GDM) diagnosed within the previous 5 years. Probands were identified from the patient populations at Los Angeles County/USC Medical Center, OB/GYN clinics at local hospitals, and the Kaiser Permanente health plan membership in Southern California. Probands qualified for participation if they: (1) were of Mexican ancestry (defined as both parents and ≥3/4 of grandparents Mexican or of Mexican descent); (2) had a confirmed diagnosis of GDM within the previous 5 years; (3) had glucose levels associated with poor pancreatic β-cell function and a high risk of diabetes when not pregnant; and (4) had no evidence of β-cell autoimmunity by GAD-65 antibody testing. Recruitment targeted two general family structures using siblings and/or first cousins of GDM probands, all with fasting glucose levels <126 mg/dl (7 mM): (1) at least two siblings and three first cousins from a single nuclear family; or (2) at least five siblings available for study. Using information from the proband to determine preliminary eligibility, siblings and first cousins were invited to participate in screening and, if eligible, detailed phenotyping (below) and collection of DNA. Available parents and connecting uncles and aunts were asked to provide DNA and had a fasting glucose determination. In addition, women of Mexican ancestry who have gone through pregnancy without GDM, as evidenced by a plasma or serum glucose level <120 mg/dl after a 50 g oral glucose screen for GDM, were also collected. Recruitment criteria for control probands were similar to that of the GDM probands, but were also selected to be age, BMI, and parity-matched to the GDM probands. Unrelated samples for the present methylation analysis were selected randomly from all BetaGene participants. The BetaGene protocol (HS-06-00045) has been approved by the Institutional Review Boards of the USC Keck School of Medicine.

#### Dataset 7: blood from East Asians and Caucasians

Here we downloaded the publicly available DNA methylation data from GSE53740 [73]. Since we found that progressive supranuclear palsy (PSP) had a significant effect on epigenetic age acceleration, we removed PSP samples from the analysis. Further, we focused on comparing East Asians to Caucasians since other racial/ethnic groups were represented by fewer than 10 samples.

#### Dataset 8: blood from African populations

We used blood methylation data from [42]. We studied peripheral whole-blood DNA from a total of 256 samples (for which the chronological age at the time of blood draw was available).

As detailed in Fagny et al. [42], the samples come from seven populations located across the Central African belt. These populations can be divided into two main groups: RHG populations, historically known as “pygmies,” who have traditionally relied on the equatorial forest for subsistence and who live close to, or within, the forest; and AGR populations, living either in rural/urban deforested regions or in forested habitats in which they practice slash-and-burn agriculture. Informed consent was obtained from all participants and from both parents of any participants under the age of 18 years. Ethical approval for this study was obtained from the institutional review boards of Institut Pasteur, France (RBM 2008-06 and 2011-54/IRB/3).

#### Dataset 9: cord blood samples from African Americans and Caucasians

These 216 cord blood samples from 92 African American and 70 Caucasian participants come from a study that described racial differences in DNA methylation levels [44].

#### Datasets 10 and 11

Saliva samples from Caucasians and Hispanics. The data were generated by splitting the data from [74] by sex, which reflected the use of these data in the development of the epigenetic clock software [20]. Note that these data were generated on the older Illumina platform (27 K array). Some of the data were used as training data in the development of the epigenetic clock, which might bias the results. By contrast, the novel saliva data from PEG (dataset 4) provide an unbiased analysis.

#### Dataset 12: lymphoblastoid cell lines from Han Chinese, African Americans, and Caucasians

We clustered the samples based on the interarray correlation. Since 51 samples were very distinct from the remaining samples, they were removed as potential outliers. Disease status did not affect the estimates of DNAm age, which is why we ignored it.

### Description of brain datasets

We collected brain datasets from six independent studies to assess gender effect on epigenetic age acceleration. We focused on Caucasian samples since there were insufficient numbers of other racial/ethnic groups.

- *Study 1: brain DNA methylation data from a study of Alzheimer’s disease study* from [75], GEO accession GSE59685. DNA methylation profiles of the cerebellum, entorhinal cortex, prefrontal cortex, and superior temporal gyrus were available from 117 individuals. We ignored disease status since it was not associated with age acceleration.
- *Study 2: brain DNA methylation data from neurologically normal participants* from [76], GEO accession GSE15745. DNA methylation data of the cerebellum, frontal cortex, pons, and temporal cortex regions from up to 148 neurologically normal participants of European ancestry [76].
- *Study 3: cerebellar DNA methylation data* from [77], GEO GSE38873. DNA methylation data from the cerebellum of 147 participants from a case-control study (121 cases/32 controls) of psychiatric disorders. Since disease status did not affect DNAm age, we ignored it.
- *Study 4: prefrontal cortex samples* from [78], GEO GSE61431. We analyzed 37 Caucasian participants (European ancestry).
- *Study 5: frontal cortex and cerebellum from neurologically normal Caucasian participants* from [79]. The DNA methylation data and corresponding SNP data can be found in dbGAP, http://www.ncbi.nlm.nih.gov/gap (accession: phs000249.v2.p1). We only analyzed 209 Caucasian participants who met our stringent quality control criteria. We excluded several putative outliers from the original dataset including three individuals who were genotyped on a different platform, six participants who were outliers according to a genetic analysis (PC plot), and 13 participants who had the wrong gender according to the gender prediction algorithm of the epigenetic clock software.
- *Study 6: dorsolateral prefrontal cortex samples from 718 Caucasian participants from the Religious Order Study (ROS) and the Memory and Aging Project (MAP)*. The DNA methylation data are available at the following webpage https://www.synapse.org/#!Synapse:syn3168763. We focused on brain samples of Caucasian participants from these two prospective cohort studies of aging that include brain donation at the time of death [80]. Additional details on the DNA methylation data can be found in [81]. We were not able to evaluate the effect of race/ethnicity on epigenetic age acceleration since the dataset contained only 12 Hispanic samples (which did not differ significantly from Caucasians in terms of epigenetic age). Further, we found no association between disease status and epigenetic age acceleration, which is why we ignored disease status in our analysis.

### Preprocessing of Illumina Infinium 450 K arrays

In brief, bisulfite conversion using the Zymo EZ DNA Methylation Kit (ZymoResearch, Orange, CA, USA) as well as subsequent hybridization of the HumanMethylation450k Bead Chip (Illumina, San Diego, CA, USA), and scanning (iScan, Illumina) were performed according to the manufacturers’ protocols by applying standard settings. DNA methylation levels (β values) were determined by calculating the ratio of intensities between methylated (signal A) and unmethylated (signal B) sites. Specifically, the β value was calculated from the intensity of the methylated (M corresponding to signal A) and unmethylated (U corresponding to signal B) sites, as the ratio of fluorescent signals β = Max(M,0)/[Max(M,0) + Max(U,0) + 100]. Thus, β values range from 0 (completely unmethylated) to 1 (completely methylated) [82]. The epigenetic clock software implements a data normalization step that repurposes the BMIQ normalization method from Teschendorff [83] so that it automatically references each sample to a gold standard based on type II probes as detailed in [20].

### Estimating blood cell counts based on DNA methylation levels

We estimate blood cell proportions using two different software tools. Houseman’s estimation method [84], which is based on DNA methylation signatures from purified leukocyte samples, was used to estimate the proportions of cytotoxic (CD8+) T cells, helper (CD4+) T, natural killer, B cells, and granulocytes. The software does not allow us to identify the type of granulocytes in blood (neutrophil, eosinophil, or basophil) but we note that neutrophils tend to be the most abundant granulocyte (~60 % of all blood cells compared with 0.5–2.5 % for eosinophils and basophils). The advanced analysis option of the epigenetic clock software [20] was used to estimate the percentage of exhausted CD8+ T cells (defined as CD28-CD45RA-) and the number (count) of naïve CD8+ T cells (defined as (CD45RA + CCR7+) as described in [31].

### Flow cytometric data from the Long Life Study of the WHI

While our DNA methylation data from the WHI were assessed at baseline, the flow cytometric data were measured 14.6 years after baseline. Between March 2012 and May 2013, a subset of WHI participants were enrolled in the Long Life Study (LLS) and additional biospecimens, physiometric, and questionnaire data were collected. All surviving Hormone Trial participants followed through 2010 and all African American and Hispanic/Latino participants from the SNP Health Association Resource (WHI-SHARe) sub-cohort were included if CVD biomarker from WHI baseline exam and genome-wide genotyping (GWAS) data were available and if they were at least 63 years old by 1 January 2012. Women who were either unable to provide informed consent (e.g. dementia) or those residing in an institution (e.g. skilled nursing facility) were excluded. Of a total of 14,081 eligible WHI participants, 9242 women consented to participate, 7875 were enrolled, and 7481 underwent successful blood draws. Blood was collected at locations across the US using a standardized protocol between March 2012 and May 2013 (Examination Management Services, Inc.) Fresh peripheral blood samples were packaged in Styrofoam with cold packs and were sent overnight to a central testing facility in Seattle.

A random sample of 600 residual fresh peripheral blood specimens (single tube, following CBC analysis) was transported to the University of Washington Medical Center’s (UWMC’s) flow cytometry laboratory and high-sensitivity, multi-parameter flow cytometry was performed utilizing a modified four-laser, multi-color Becton-Dickinson (BD; San Jose, CA, USA) LSRII flow cytometer. All of the flow cytometry studies were performed within 72 h of sample collection between June 2012 and February 2013. A single tube was used to evaluate T lymphocyte subsets: CD45 (KO), CD8 (BV), CD45RA (F), CCR7 (PE), CD5 (ECD), CD56 (PC5), CD3 (APC-H7), CD4 (A594), CD28 (APC), CD27 (PC7). A second tube evaluated B lymphocyte subsets: CD45 (APC-H7), CD20 (V450), kappa (F), lambda (PE), CD23 (ECD), CD5 (PC5.5), CD19 (BV650), CD38 (A594), CD10 (APC), CD27 (PC7), CD3 (APC-A700). Categories of circulating cells were quantified using a predefined population-based gating strategy based on established gating strategies for both T lymphocyte [85] and B lymphocyte [86] subsets.

### Flow cytometric data from the MACS cohort

As part of Additional file 2, we validated imputed blood cell counts using flow cytometric data and DNA methylation data collected from men of the Multi-Center AIDS Cohort Study (MACS). The data were generated as described in [87]. Briefly, human peripheral blood mononuclear cell (PBMC) samples were isolated from fresh blood samples and either stained for flow cytometry analysis or used for genomic DNA isolation. DNA was isolated from 1 × 10^6^ PBMC using Qiagen DNeasy blood and tissue mini spin columns. Quality of DNA samples was assessed using Nanodrop measurements and accurate DNA concentrations were measured using a Qubit assay kit (Life Technology). Cryopreserved PBMC obtained from the repository were thawed and assayed for viability using trypan blue. The mean viability of the samples was 88 %. Samples were stained for 30 min at 4 °C with the following antibody combinations of fluorescently conjugated monoclonal antibodies using the manufacturers recommended amounts for 1 million cells: tube 1: CD57 FITC (clone HNK-1), CD28 phycoerythrin (PE, L293), CD3 peridinin chlorophyll protein (PerCP,SK7), CD45RA phycoerythrin cyanine dye Cy7 tandem (PE-Cy7, L48), CCR7 Alexa Fluor 647 (AF647, 150503), CD8 allophycocyanin H7- tandem (APC-H7, SK1) and CD4 horizon V450 (V450, RPA-T4); tube 2: HLA-DR FITC (L243), CD38 PE (HB7), CD3 PercP, CD45RO PE-Cy7 (UCHL-1), CD95-APC(DXZ), CD8 APC-H7, and CD4 V450); tube 3: CD38 FITC (HB7), IgD PE (1A6–2), CD3 PerCP, CD10 PE-Cy7 (HI10a), CD27 APC (eBioscience, clone 0323, San Diego, CA), CD19 APC-H7 (SJ25C1) and CD20 V450 (L27). Antibodies were purchased from BD Biosciences, San Jose, CA (BD) except as noted. Stained samples were washed twice with staining buffer and run immediately on an LSR2 cytometer equipped with a UV laser (BD, San Jose, CA, USA) for the detection of 4′,6-diamidino-2-phenylindole dihydrochloride (DAPI) which was used as a viability marker at a final concentration of 0.1 ug/mL. Lineage gated isotype controls to measure non-specific binding were run and used CD3, CD4, and CD8 for T-cells or CD19 for B-cells. Fluorescence minus one controls (FMO) were also utilized to assist gating and cursor setting. A range of 20,000–100,000 lymphocytes were acquired and analyzed per sample using the FACSDiva software package (BD, San Jose, CA, USA).

## Additional files

Additional file 1: Lymphoblastoid cell lines from Han Chinese, Caucasians, and African Americans. **A** *Gray line* corresponds to a natural spline regression through Caucasian samples. Age acceleration was defined as residual with respect to this line. **B** Marginally significant evidence that African American’s are younger than other ethnic groups. (PDF 33 kb) Additional file 2: Accuracy of imputed blood cell counts. Here we used an independent dataset, which was not used to develop estimators of blood cell counts based on DNA methylation data, to evaluate the accuracy of the imputed blood cell counts. For each participant, both flow cytometric measures and Illumina Inf450 data were available from 96 participants as described in [88]. **A**-**G** The *scatter plots* depict the predicted abundance of blood cell count (based on DNA methylation levels) versus the corresponding observed flow cytometric measurement (*y-axis*). Each panel reports a robust correlation coefficient (biweight midcorrelation) and a corresponding *p* value. The Houseman method was used to impute (**A**) CD8+ T cells, (**B**) CD4+ T, (**C**) B cells. The epigenetic clock software was used for imputing (**D**) naïve CD8+ T cells, (**E**) naïve CD4 + T cells, (**F**) plasma blasts, and (**G**) exhausted CD8+ T cells. **H**, **I** Another flow cytometric dataset was used to test for ethnic differences in naïve CD4+ T cells. The *y-axis* shows the log transformed flow cytometric measurement of naïve CD4+ T cells (adjusted for age). Specifically, the *y-axis* reports the residual resulting from regressing log(naïve CD4+ T cell abundance) on chronological age. **H** Findings for HIV– participants (198 Caucasians versus 34 Hispanics). **I** Findings for HIV+ participants (101 Caucasians, 58 Hispanics). Stouffer’s meta-analysis across the two strata (HIV+ and HIV– strata) shows that Hispanics have significantly fewer naïve CD4+ T cells (Stouffer’s *p* = 0.030, Stouffer’s Z = (1.75 + 1.31)/sqrt(2)). (PDF 130 kb) Additional file 3: Epigenetic age acceleration in Hispanics versus country of residence in the WHI. Each *column* corresponds to different measure of age acceleration: (**A**, **D**) age acceleration residual, (**B**, **E**) IEAA (**C**, **F**) EEAA. (**A**-**C**, *first row*) results for “country of birth” (*x-axis*). (**D**-**F**, *second row*) results for “country of residence” at age 35 years, which was defined by combining two variables country of birth and “living in the US at age 35.” The *left-most bar* corresponds to Hispanic women who were born outside the US and lived outside the US at age 35 years, the *middle bar* corresponds to Hispanic women who were born outside the US but lived already in the US at the age of 35 years; the *right-most bar* reports results for women who were born in the US and lived in the US at age 35 years. Incidentally, all of these postmenopausal Hispanic women lived in the US at the age of the blood draw. As a caveat, we mention the relatively small group sizes (*small gray numbers underneath the bars*). (PDF 3 kb) Additional file 4: Educational level versus age acceleration in the WHI. Each *row* relates educational level (*x-axis*) to three respective measures of epigenetic age acceleration: (**A**-**D**) Age Accel., (**E**-**H**) IEAA, and (**I**-**L**) EEAA. The columns correspond to different groups of women from the WHI. The first, second, third, and fourth *columns* report findings for (**A**, **E**, **I**) all women, (**B**, **F**, **J**) Caucasians, (**C**, **G**, **K**) African Americans, and (**D**, **H**, **L**) Hispanics, respectively. Each *bar plot* reports the mean values, 1 standard error, and the *p* value from a non-parametric group comparison test (Kruskal–Wallis). Education was assessed using the form “Demographics and Study Membership.” We find that education predicts future EEAA. (PDF 6 kb) Additional file 5: Hypertension status versus age acceleration in the Bogalusa study. Each *row* relates hypertension status (*x-axis*) to three respective measures of epigenetic age acceleration: (**A**-**C**) Age Accel., (**D**-**F**) IEAA, and (**G**-**I**) EEAA. The columns correspond to different groups. The first, second, and third *columns* report findings for (**A**, **D**, **G**) all participants, (**B**, **E**, **H**) Caucasians, (**C**, **F**, **I**) African Americans, respectively. Each *bar plot* reports the mean values, 1 standard error, and the *p* value from a non-parametric group comparison test (Kruskal–Wallis). Hypertension status was defined as meeting any of the three conditions: (1) blood pressure > =140/90; (2) taking medication; or (3) having been diagnosed as having hypertension. (PDF 4 kb) Additional file 6: Type II diabetes status versus age acceleration in the Bogalusa study. Each *row* relates type II diabetes status (*x-axis*) to three respective measures of epigenetic age acceleration: (**A**-**C**) Age Accel., (**D**-**F**) IEAA, and (**G**-**I**) EEAA. The *columns* correspond to different groups. The first, second, and third *columns* report findings for (**A**, **D**, **G**) all participants, (**B**, **E**, **H**) Caucasians, (**C**, **F**, **I**) African Americans, respectively. Each *bar plot* reports the mean values, 1 standard error, and the *p* value from a non-parametric group comparison test (Kruskal–Wallis). Type 2 diabetes status was defined as fasting glucose > =126 mg/dl or taking diabetes medication. (PDF 3 kb) Additional file 7: Educational level versus age acceleration in the Bogalusa study. Each *row* relates educational level (*x-axis*) to three respective measures of epigenetic age acceleration: (**A**-**C**) Age Accel., (**D**-**F**) IEAA, and (**G**-**I**) EEAA. The *columns* correspond to different groups. The first, second, and third *columns* report findings for (**A**, **D**, **G**) all participants, (**B**, **E**, **H**) Caucasians, (**C**, **F**, **I**) African Americans, respectively. Each *bar plot* reports the mean values, 1 standard error, and the *p* value from a non-parametric group comparison test (Kruskal–Wallis). Education was grouped as follows: group 1 = grades 1–7; group 2 = grades 8–9; group 3 = grades 10–12; group 4 = vocational/tech training; group 5 = college; group 6 = postgraduate. (PDF 5 kb) Additional file 8: Robustness analysis with respect to other epigenetic biomarkers of aging in the WHI. **A**-**C** Results for the Horvath method when 47 out of 353 CpGs were removed from the epigenetic clock (because they are in the vicinity of a SNP). Since none of the remaining clock CpGs are near a SNP, the resulting age acceleration is not trivially related to race/ethnicity. **A** DNA methylation age versus chronological age. **B** Ethnicity versus age acceleration (defined as residual resulting from regressing DNAm age on chronological age). **C** Intrinsic epigenetic age acceleration versus ethnicity. **D**, **E** Alternative epigenetic biomarker of aging based on 589 age-related CpGs from Teschendorff [13]. The biomarker was defined using the following steps. First, the DNA methylation levels of each CpGs were standardized (to mean zero and variance 1). Second, a weighted average was formed by multiplying each CpG by the T test statistic from the chronological age relationship based on the table from the original reference. Third, the weighted average was regressed on chronological age to arrive at a residual. The resulting residual is referred to as extrinsic measure of age acceleration since it was not adjusted for blood cell counts. Fourth, the resulting measure was regressed on estimated blood cell counts (analogous to those used for IEAA) in order to arrive an intrinsic measure of age acceleration. **F**, **G** Epigenetic measures of age acceleration using the Hannum method 71 CpGs [19]. **D**, **F** Results for intrinsic measures, i.e. measures of age acceleration that adjust both for blood cell counts and chronological age. **E**, **G** reports extrinsic measures, i.e. no adjustment for imputed blood cell counts. Each *bar plot* depicts 1 standard error and reports the results from a Kruskal–Wallis test. (PDF 55 kb) Additional file 9: Demographic and physiologic characteristics of women from the WHI. Case-control status refers to CHD. Two designs were used to select samples: case/control and case-cohort. (DOC 48 kb)
