# Supplementary figures and images for: An epigenetic clock analysis of race/ethnicity, sex, and coronary heart disease
Source: Genome Biol. 2016 Aug 11;17:171. doi: 10.1186/s13059-016-1030-0 (PMC4980791; doi:10.1186/s13059-016-1030-0)

**A** Non outlying  $\text{cor}=0.59$ ,  $p=1.3\text{e-}23$

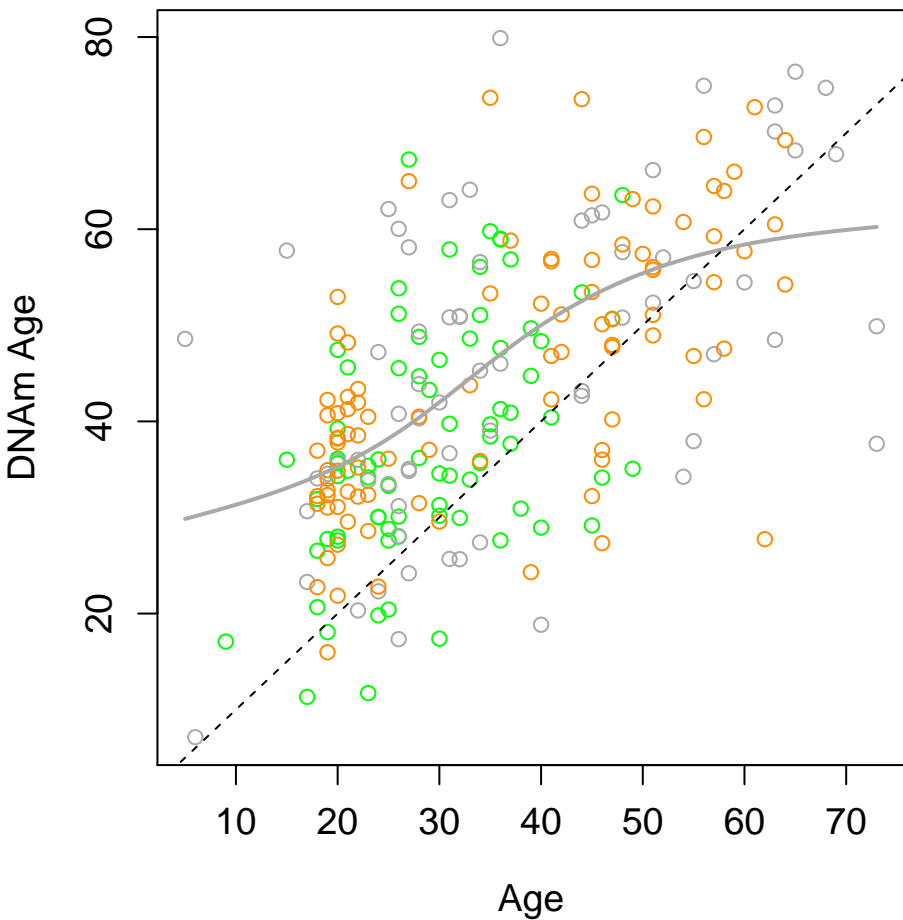

**B** Non outlying  $p = 0.073$

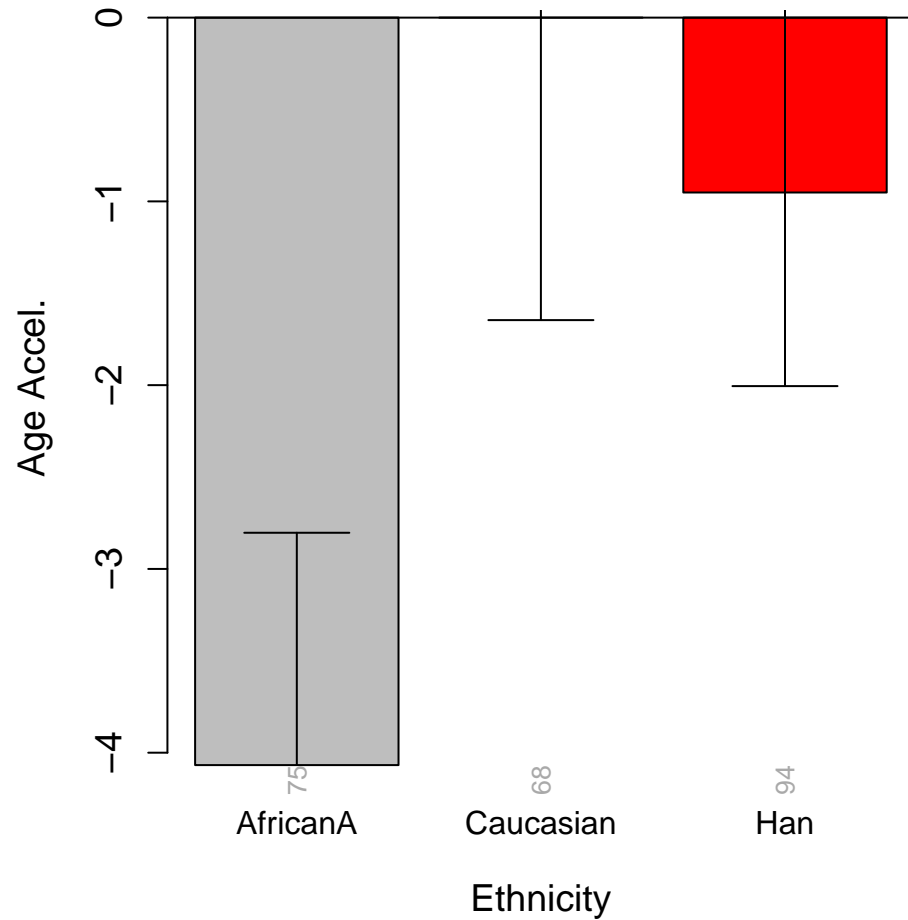

Supplement: Additional file 1: — Lymphoblastoid cell lines from Han Chinese, Caucasians, and African Americans. A Gray line corresponds to a natural spline regression through Caucasian samples. Age acceleration was defined as residual with respect to this line. B Marginally significant evidence that African American’s are younger than other ethnic groups. (PDF 33 kb) [file 13059_2016_1030_MOESM1_ESM.pdf]

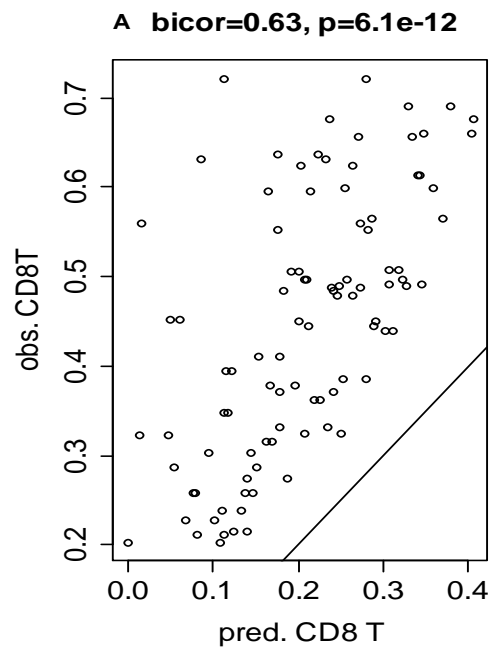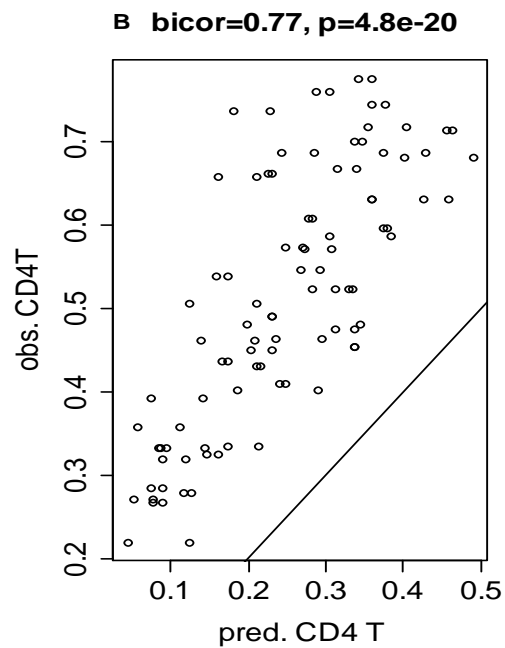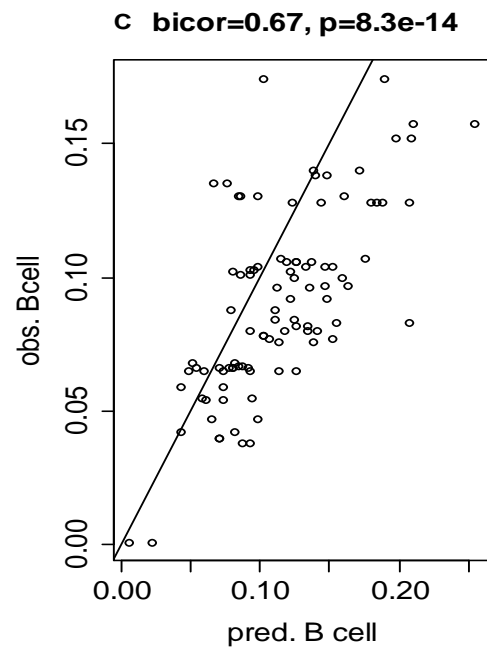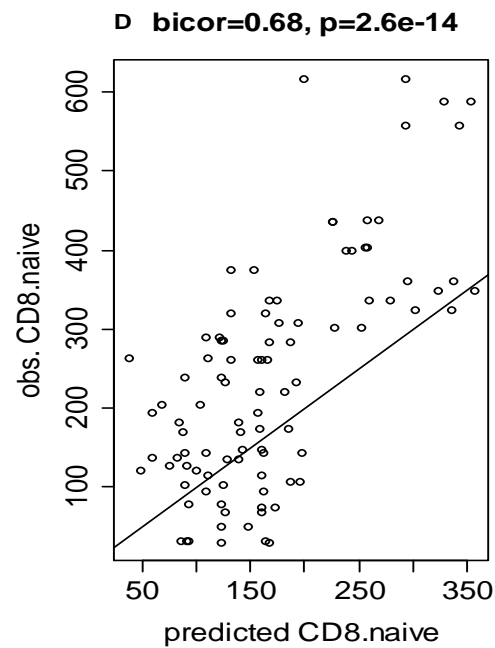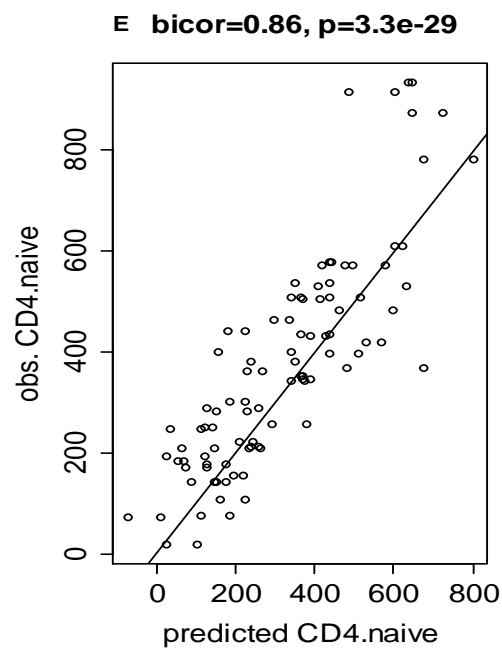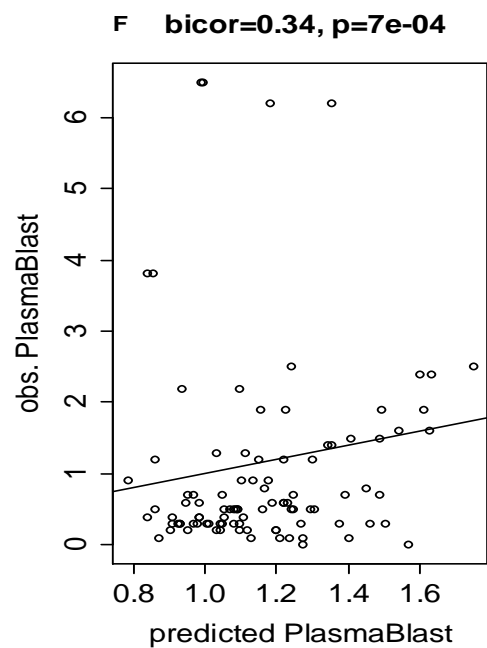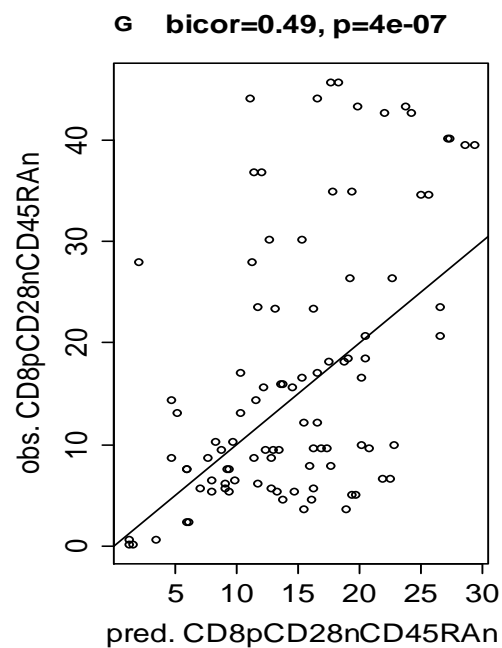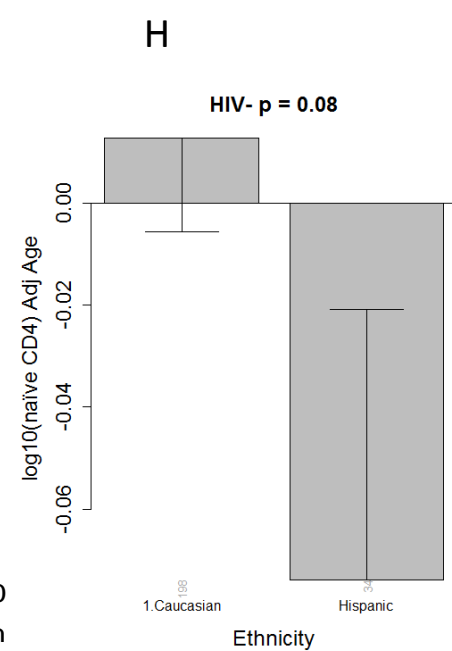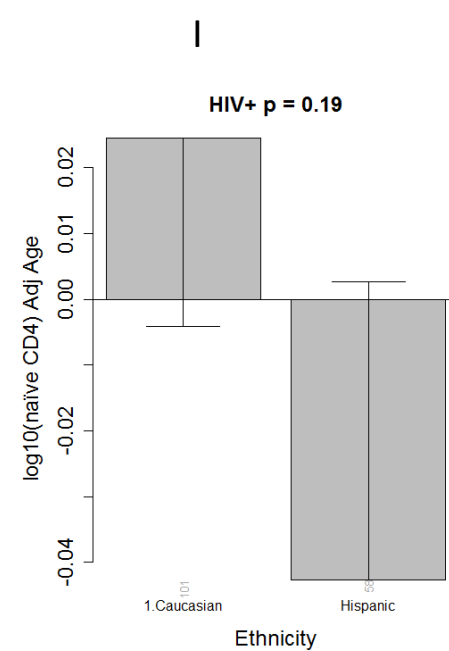

Supplement: Additional file 2: — Accuracy of imputed blood cell counts. Here we used an independent dataset, which was not used to develop estimators of blood cell counts based on DNA methylation data, to evaluate the accuracy of the imputed blood cell counts. For each participant, both flow cytometric measures and Illumina Inf450 data were available from 96 participants as described in [88]. A-G The scatter plots depict the predicted abundance of blood cell count (based on DNA methylation levels) versus the corresponding observed flow cytometric measurement (y-axis). Each panel reports a robust correlation coefficient (biweight midcorrelation) and a corresponding p value. The Houseman method was used to impute (A) CD8+ T cells, (B) CD4+ T, (C) B cells. The epigenetic clock software was used for imputing (D) naïve CD8+ T cells, (E) naïve CD4 + T cells, (F) plasma blasts, and (G) exhausted CD8+ T cells. H, I Another flow cytometric dataset was used to test for ethnic differences in naïve CD4+ T cells. The y-axis shows the log transformed flow cytometric measurement of naïve CD4+ T cells (adjusted for age). Specifically, the y-axis reports the residual resulting from regressing log(naïve CD4+ T cell abundance) on chronological age. H Findings for HIV– participants (198 Caucasians versus 34 Hispanics). I Findings for HIV+ participants (101 Caucasians, 58 Hispanics). Stouffer’s meta-analysis across the two strata (HIV+ and HIV– strata) shows that Hispanics have significantly fewer naïve CD4+ T cells (Stouffer’s p = 0.030, Stouffer’s Z = (1.75 + 1.31)/sqrt(2)). (PDF 130 kb) [file 13059_2016_1030_MOESM2_ESM.pdf]

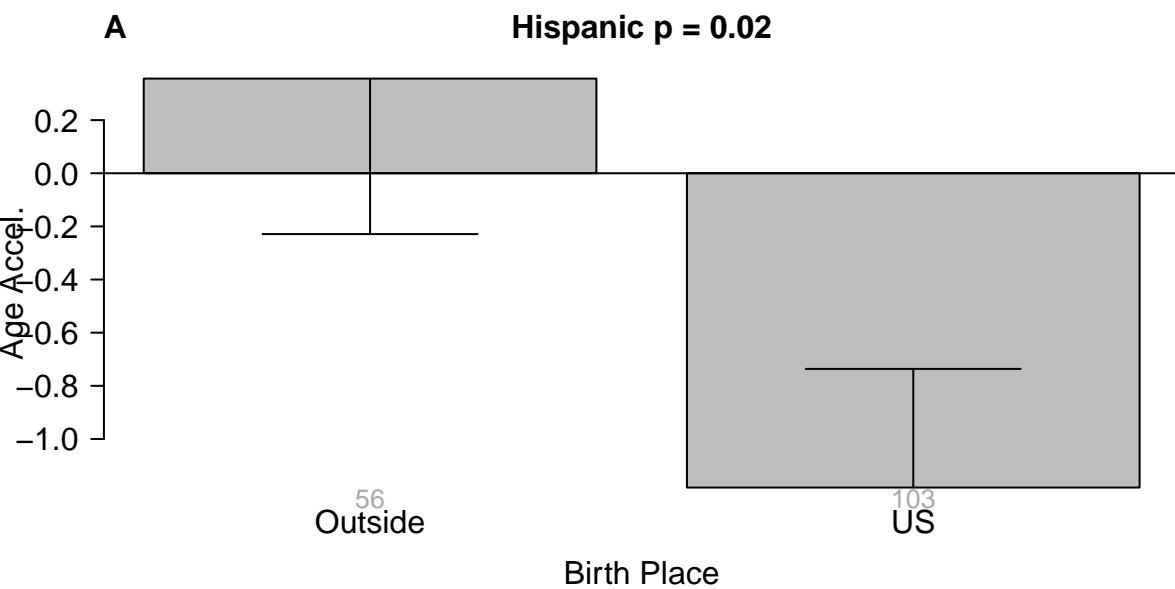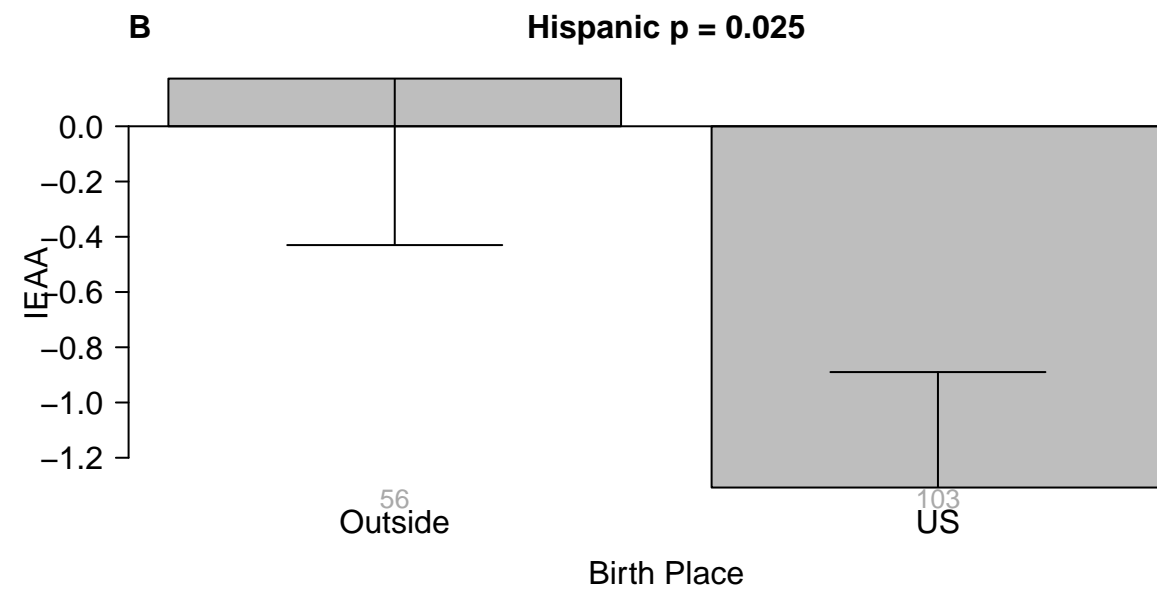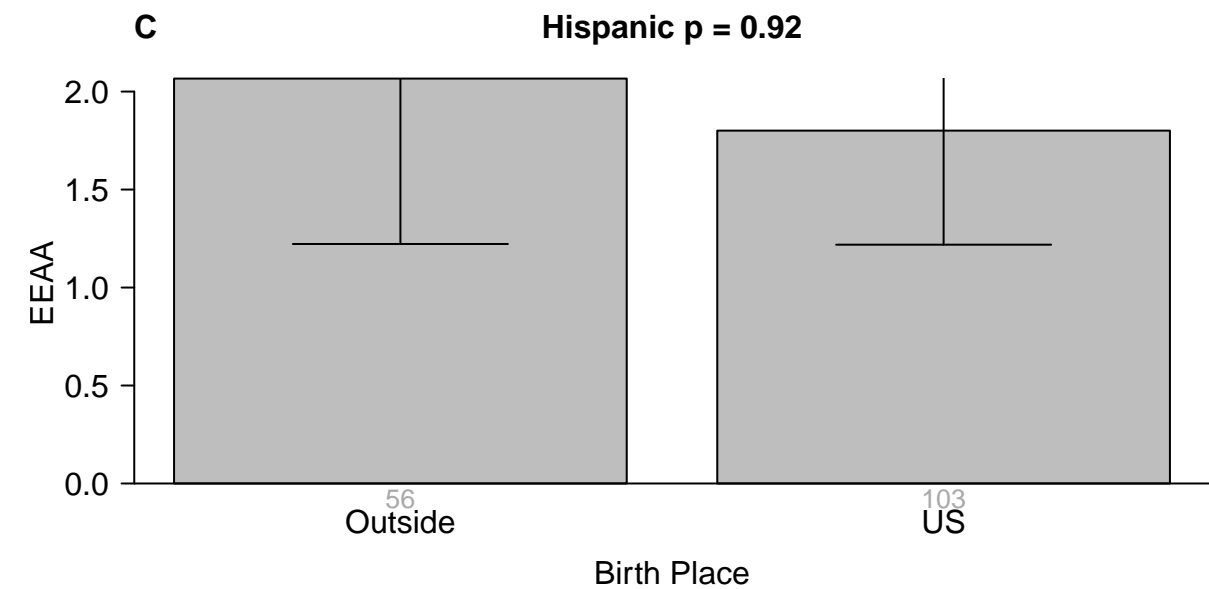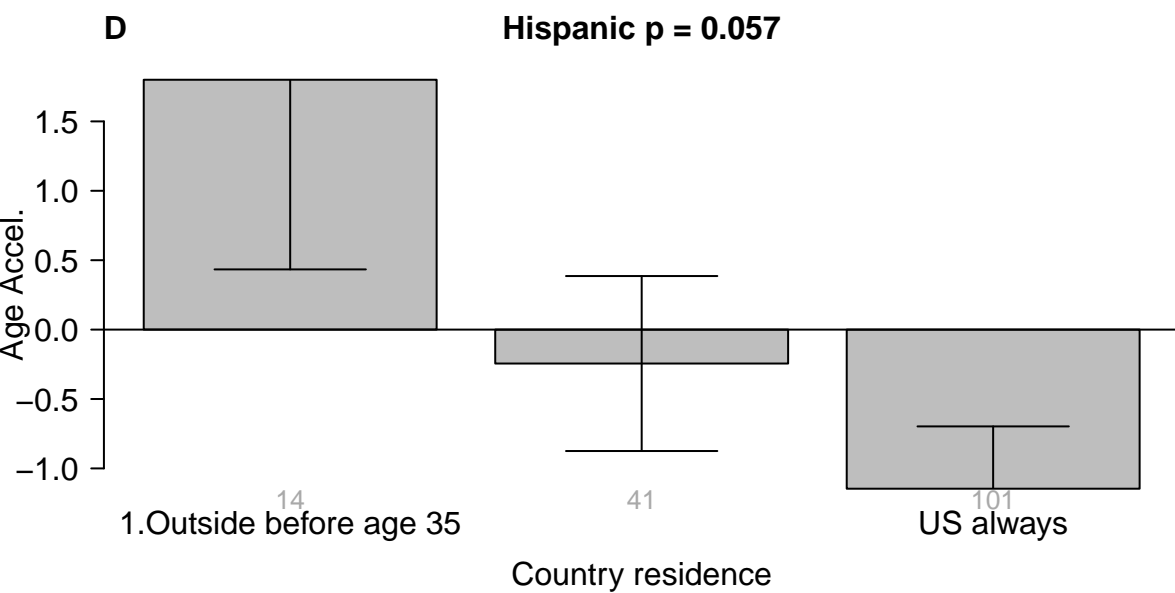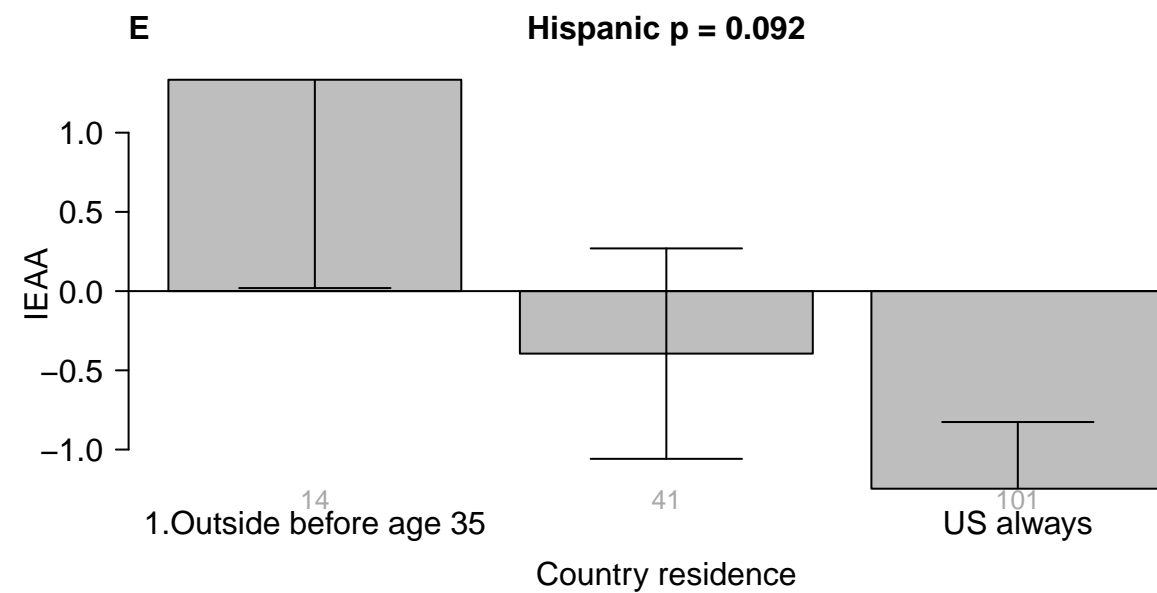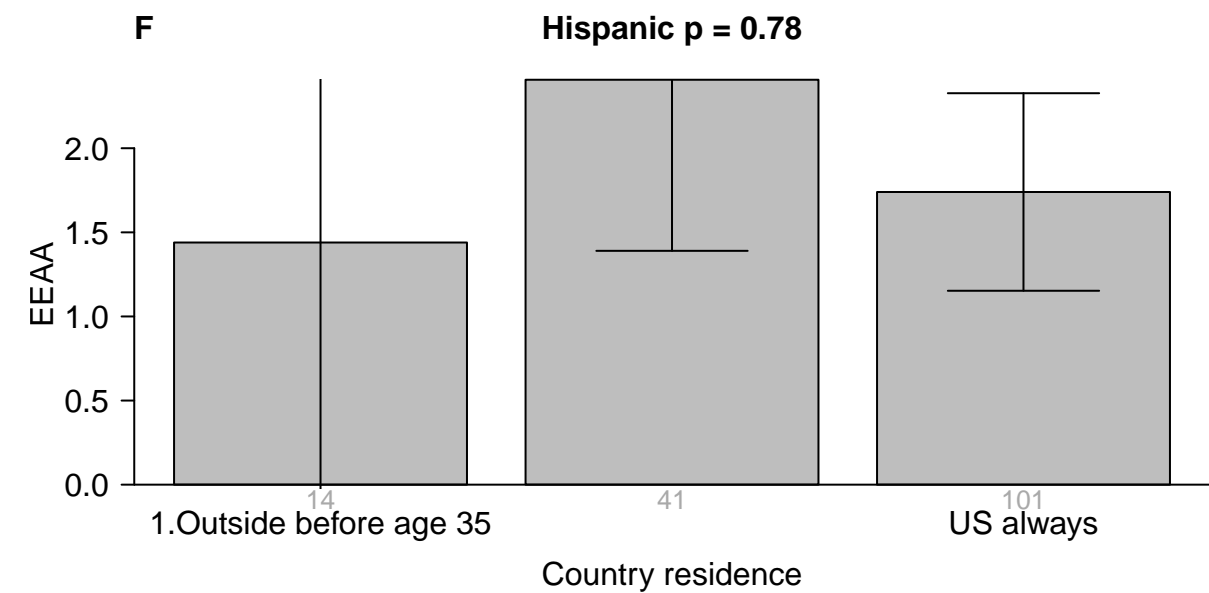

Supplement: Additional file 3: — Epigenetic age acceleration in Hispanics versus country of residence in the WHI. Each column corresponds to different measure of age acceleration: (A, D) age acceleration residual, (B, E) IEAA (C, F) EEAA. (A-C, first row) results for “country of birth” (x-axis). (D-F, second row) results for “country of residence” at age 35 years, which was defined by combining two variables country of birth and “living in the US at age 35.” The left-most bar corresponds to Hispanic women who were born outside the US and lived outside the US at age 35 years, the middle bar corresponds to Hispanic women who were born outside the US but lived already in the US at the age of 35 years; the right-most bar reports results for women who were born in the US and lived in the US at age 35 years. Incidentally, all of these postmenopausal Hispanic women lived in the US at the age of the blood draw. As a caveat, we mention the relatively small group sizes (small gray numbers underneath the bars). (PDF 3 kb) [file 13059_2016_1030_MOESM3_ESM.pdf]

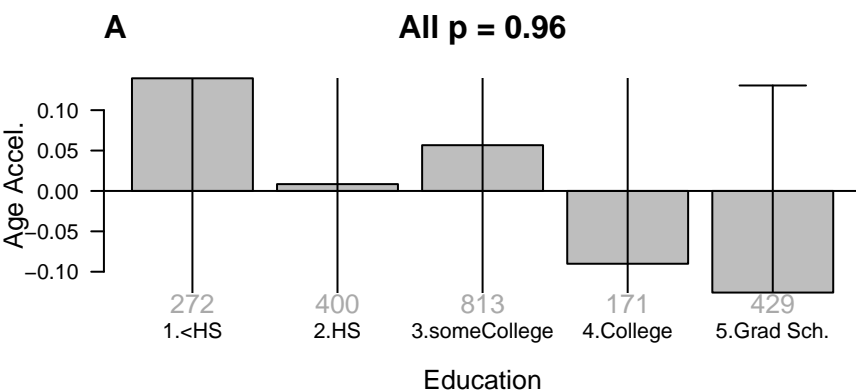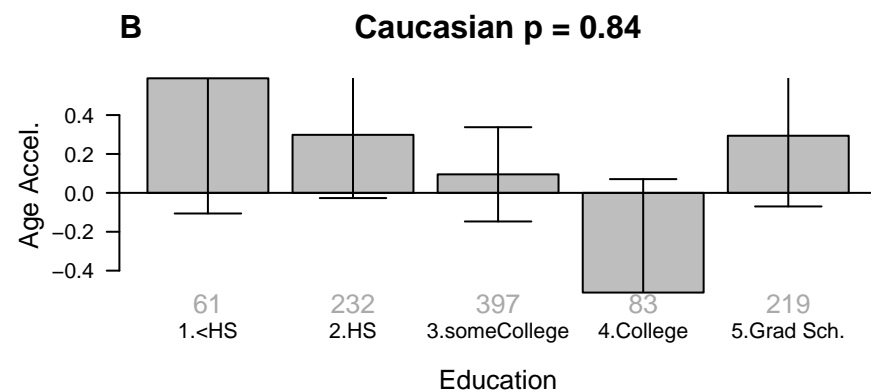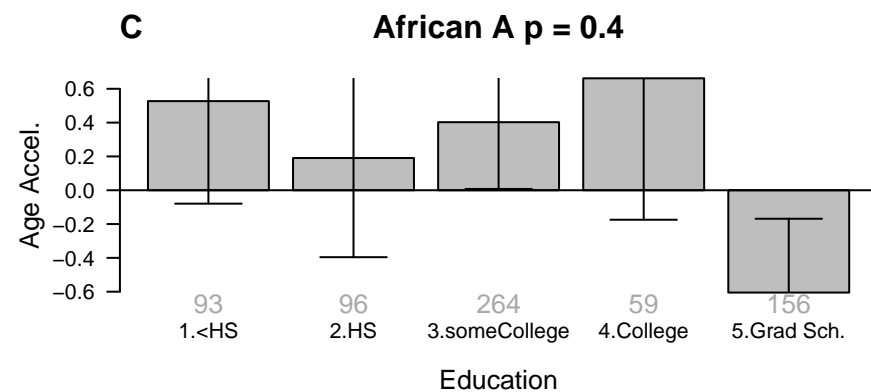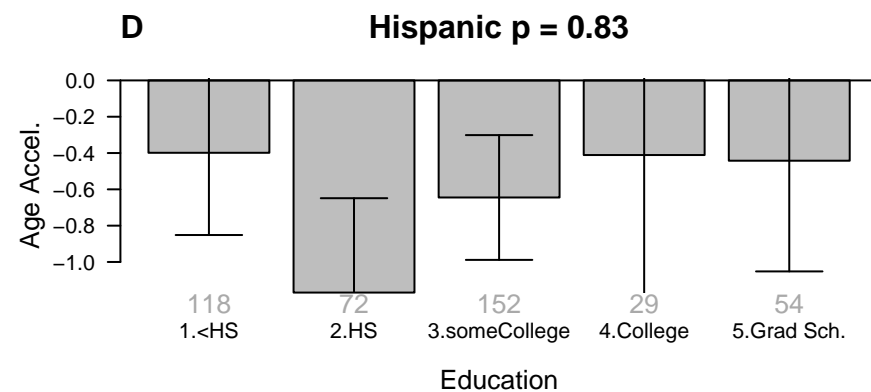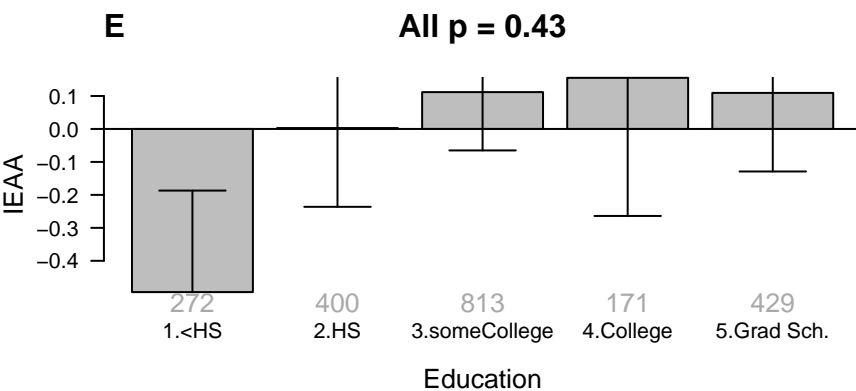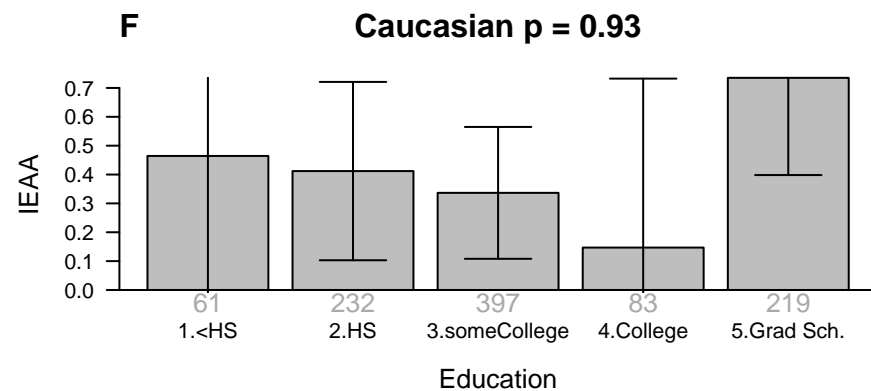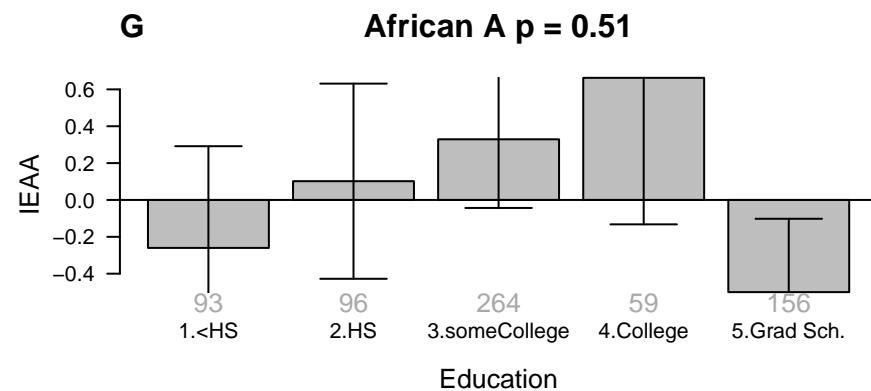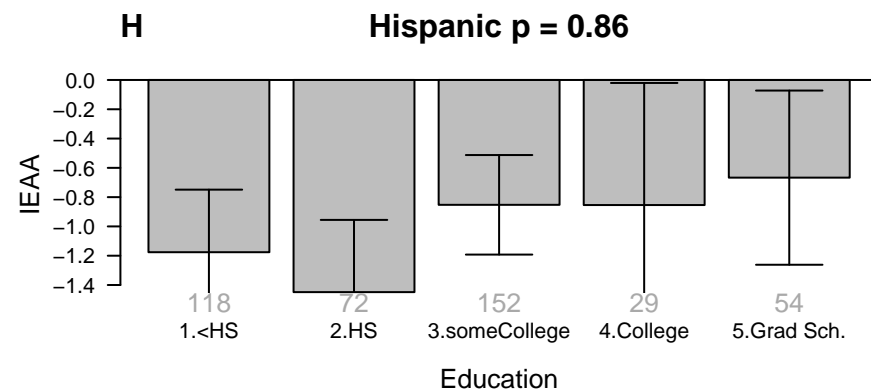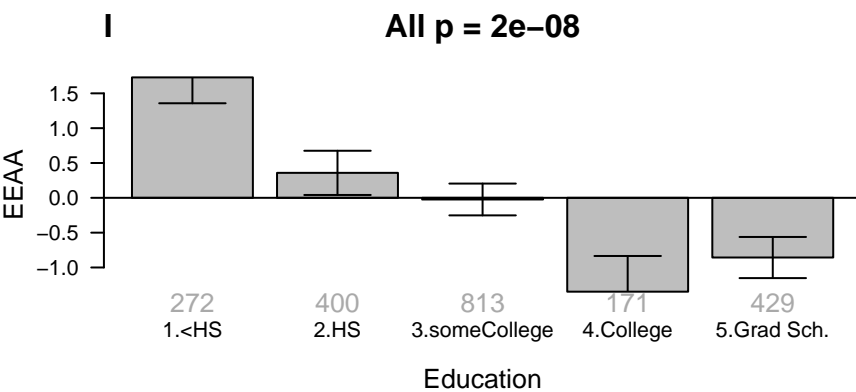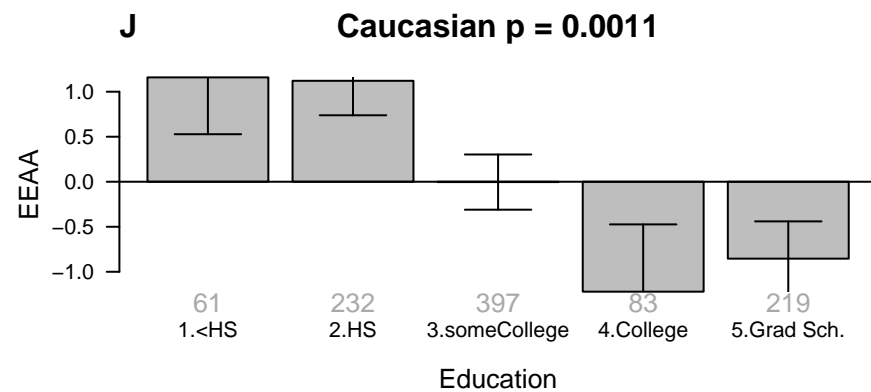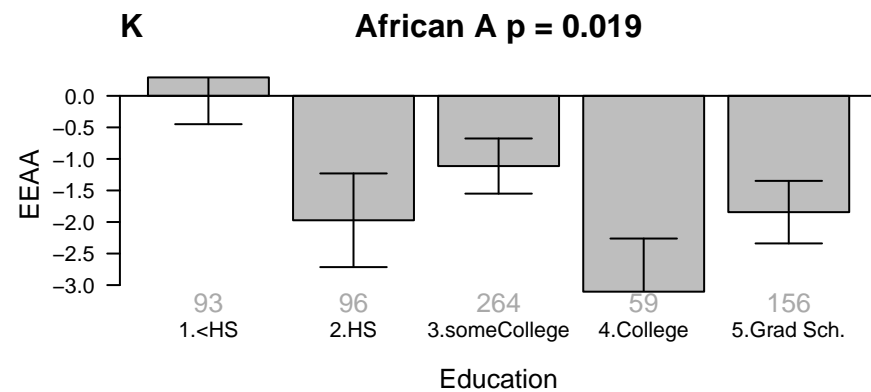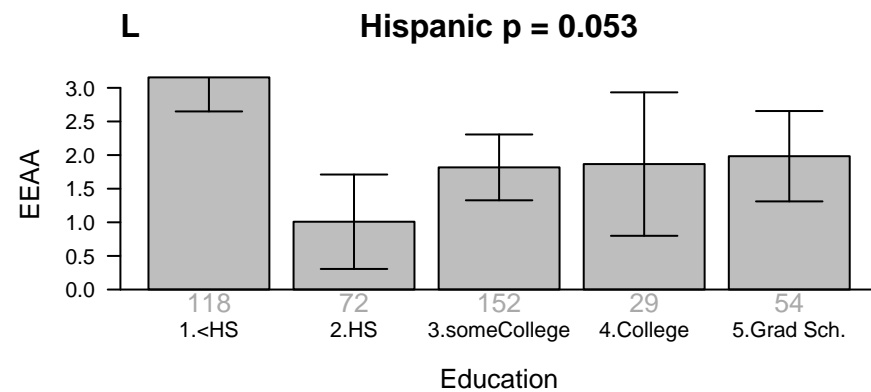

Supplement: Additional file 4: — Educational level versus age acceleration in the WHI. Each row relates educational level (x-axis) to three respective measures of epigenetic age acceleration: (A-D) Age Accel., (E-H) IEAA, and (I-L) EEAA. The columns correspond to different groups of women from the WHI. The first, second, third, and fourth columns report findings for (A, E, I) all women, (B, F, J) Caucasians, (C, G, K) African Americans, and (D, H, L) Hispanics, respectively. Each bar plot reports the mean values, 1 standard error, and the p value from a non-parametric group comparison test (Kruskal–Wallis). Education was assessed using the form “Demographics and Study Membership.” We find that education predicts future EEAA. (PDF 6 kb) [file 13059_2016_1030_MOESM4_ESM.pdf]

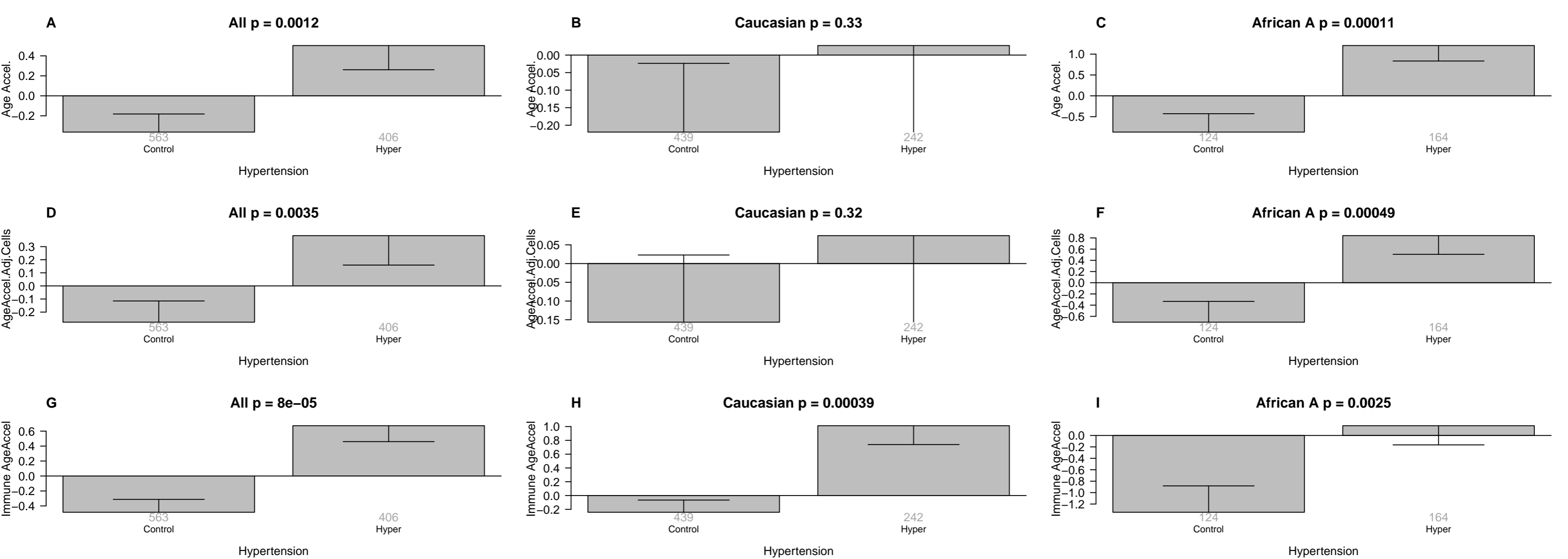

Supplement: Additional file 5: — Hypertension status versus age acceleration in the Bogalusa study. Each row relates hypertension status (x-axis) to three respective measures of epigenetic age acceleration: (A-C) Age Accel., (D-F) IEAA, and (G-I) EEAA. The columns correspond to different groups. The first, second, and third columns report findings for (A, D, G) all participants, (B, E, H) Caucasians, (C, F, I) African Americans, respectively. Each bar plot reports the mean values, 1 standard error, and the p value from a non-parametric group comparison test (Kruskal–Wallis). Hypertension status was defined as meeting any of the three conditions: (1) blood pressure > =140/90; (2) taking medication; or (3) having been diagnosed as having hypertension. (PDF 4 kb) [file 13059_2016_1030_MOESM5_ESM.pdf]

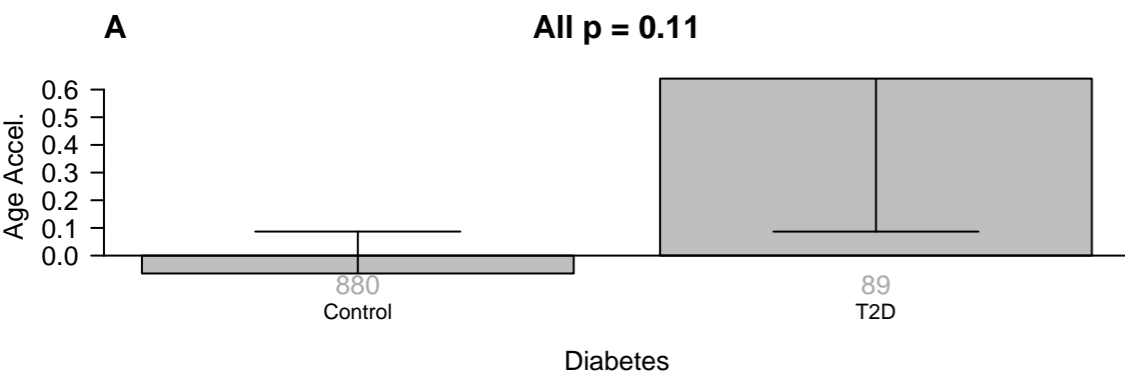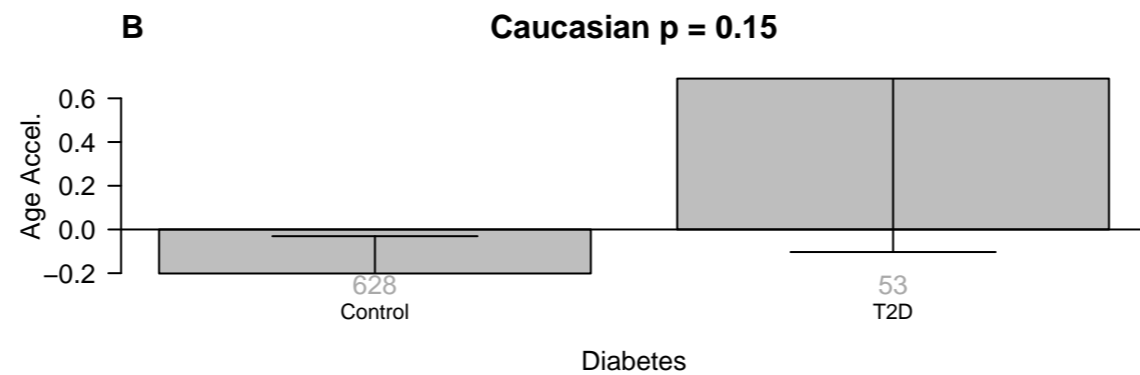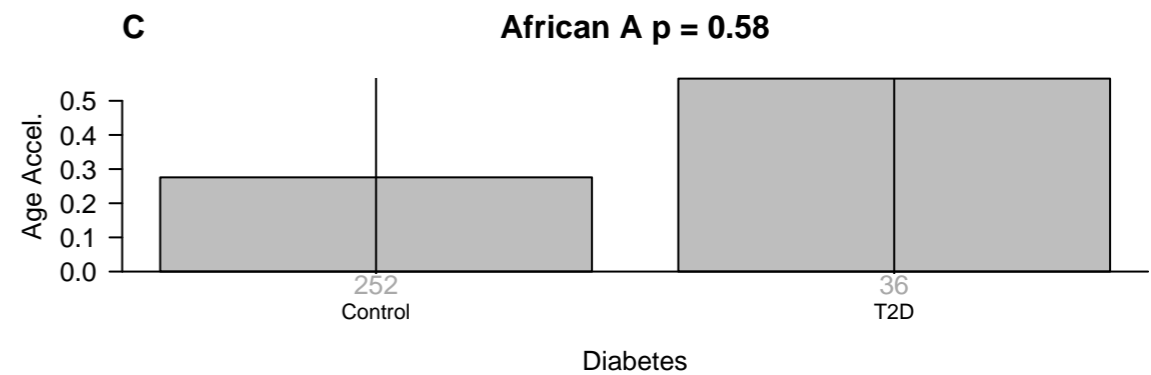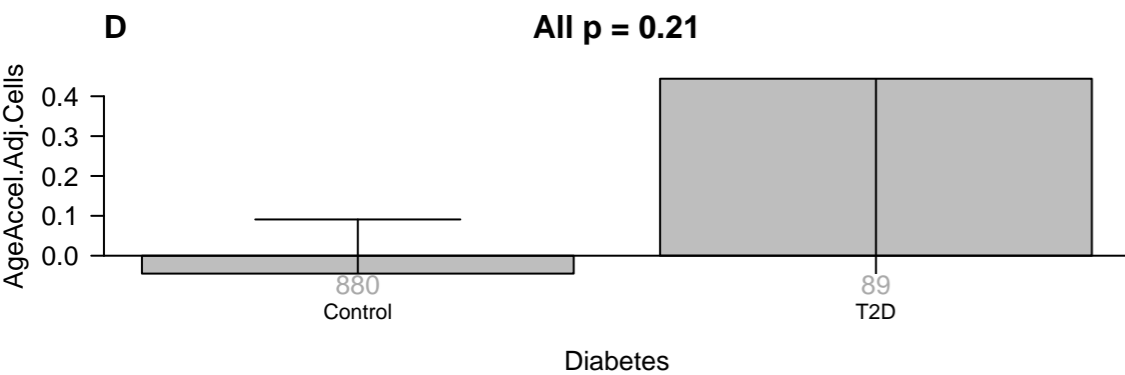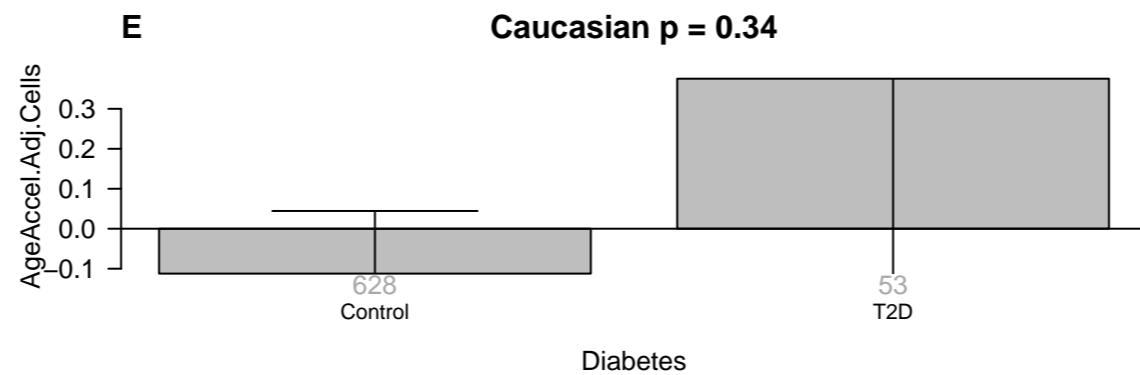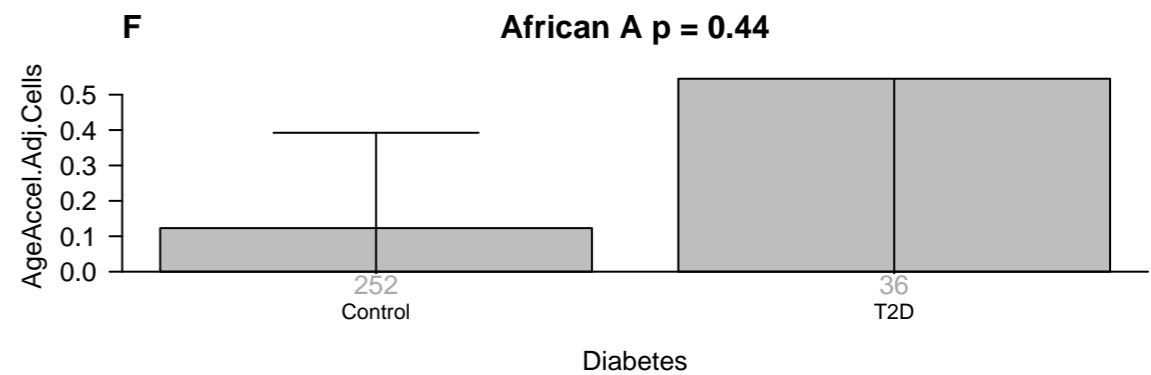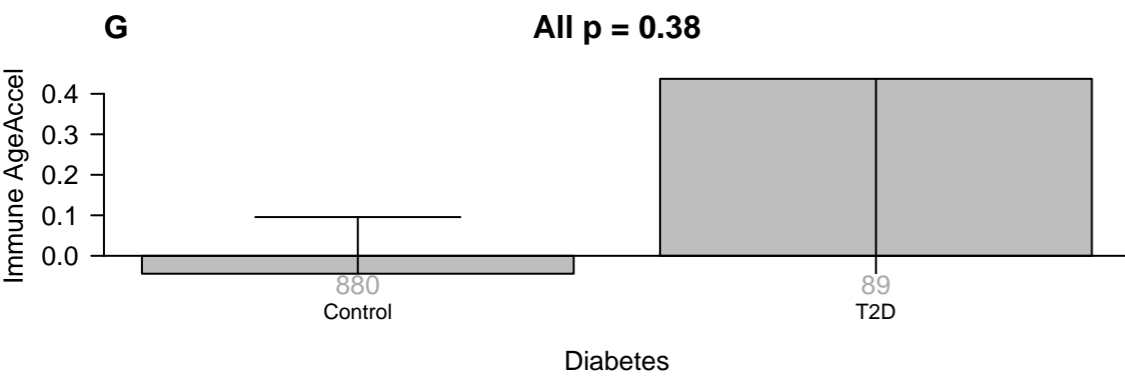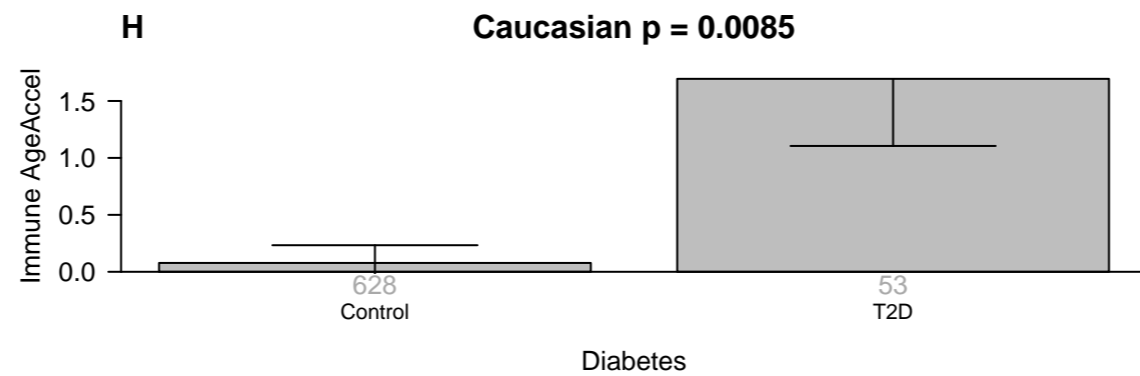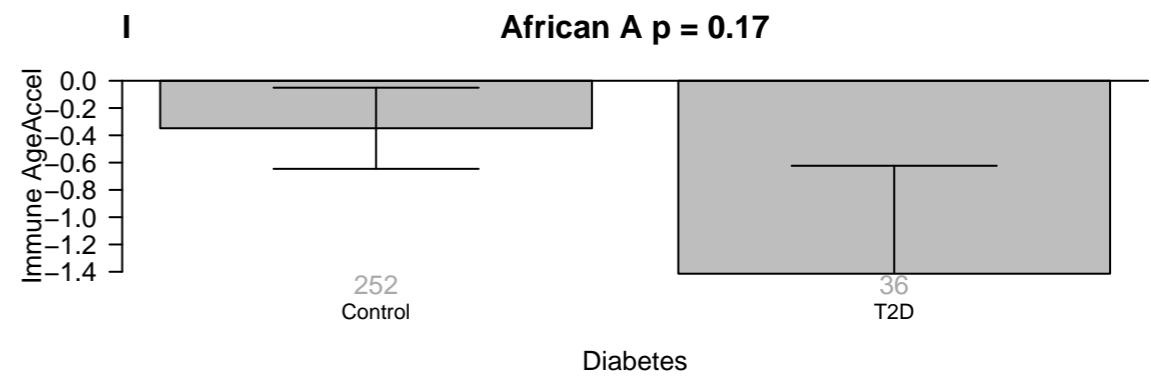

Supplement: Additional file 6: — Type II diabetes status versus age acceleration in the Bogalusa study. Each row relates type II diabetes status (x-axis) to three respective measures of epigenetic age acceleration: (A-C) Age Accel., (D-F) IEAA, and (G-I) EEAA. The columns correspond to different groups. The first, second, and third columns report findings for (A, D, G) all participants, (B, E, H) Caucasians, (C, F, I) African Americans, respectively. Each bar plot reports the mean values, 1 standard error, and the p value from a non-parametric group comparison test (Kruskal–Wallis). Type 2 diabetes status was defined as fasting glucose > =126 mg/dl or taking diabetes medication. (PDF 3 kb) [file 13059_2016_1030_MOESM6_ESM.pdf]

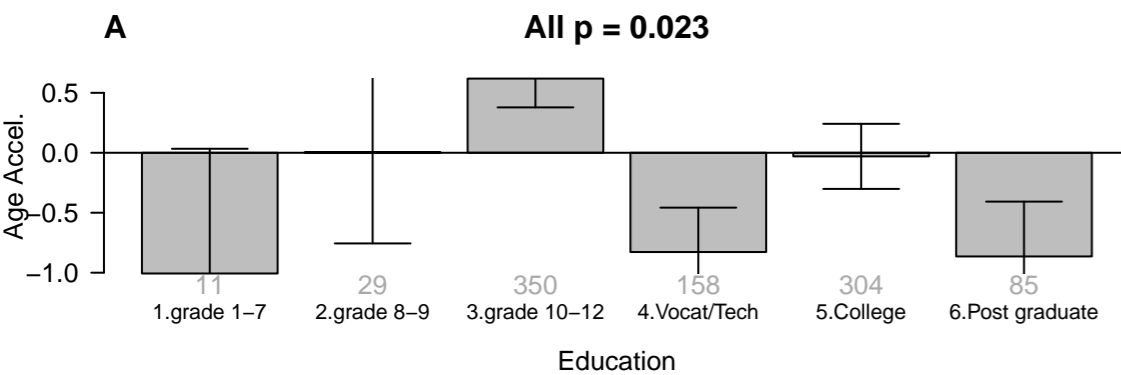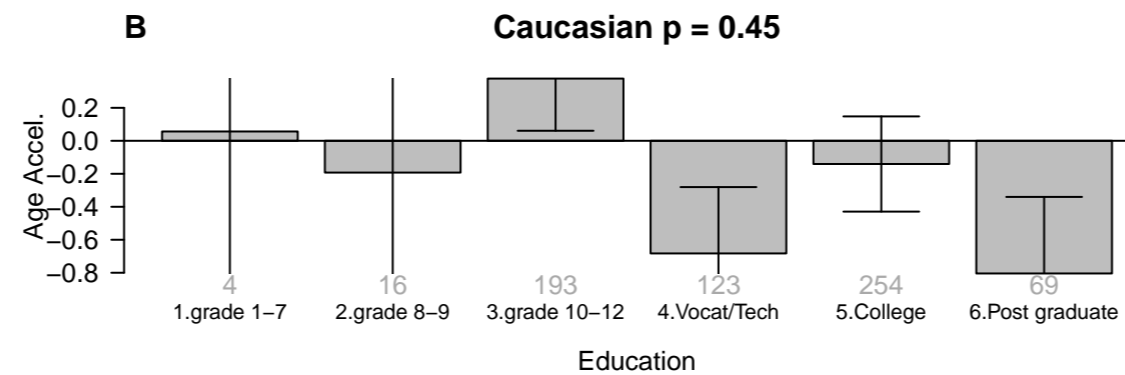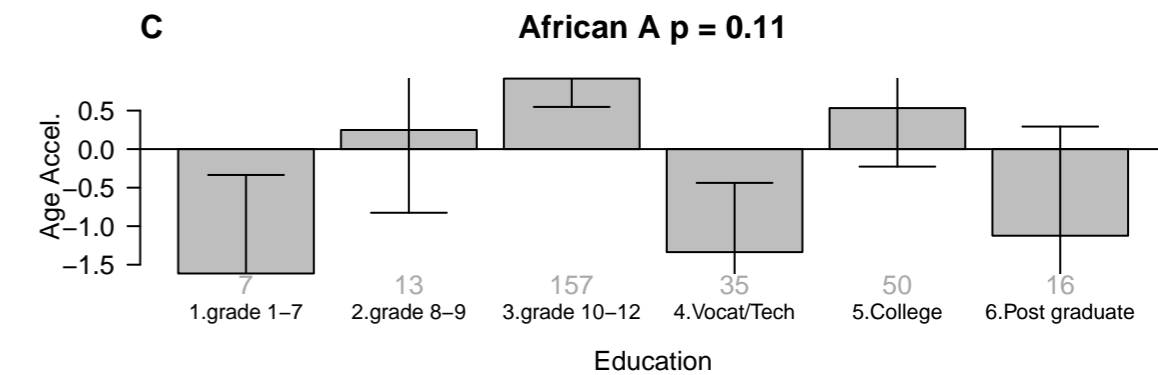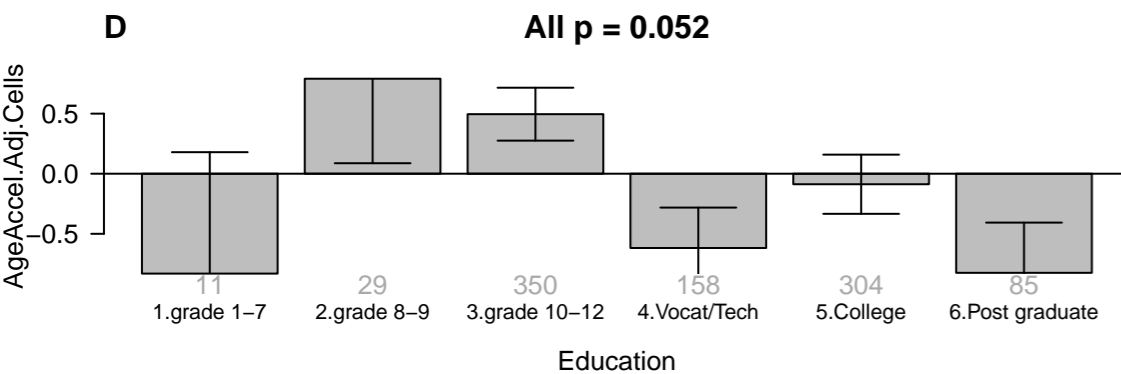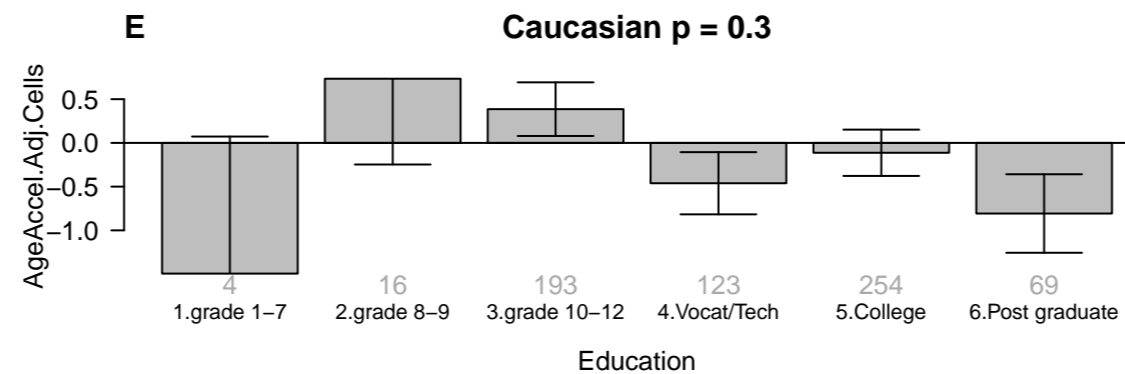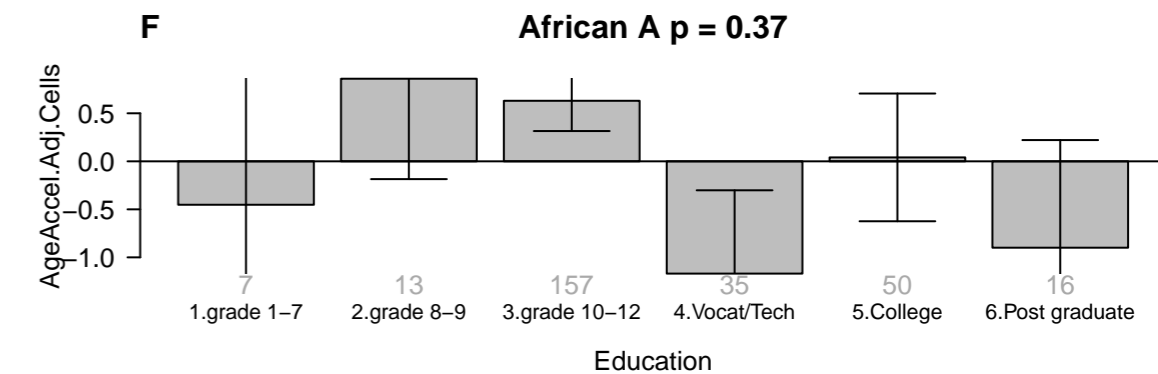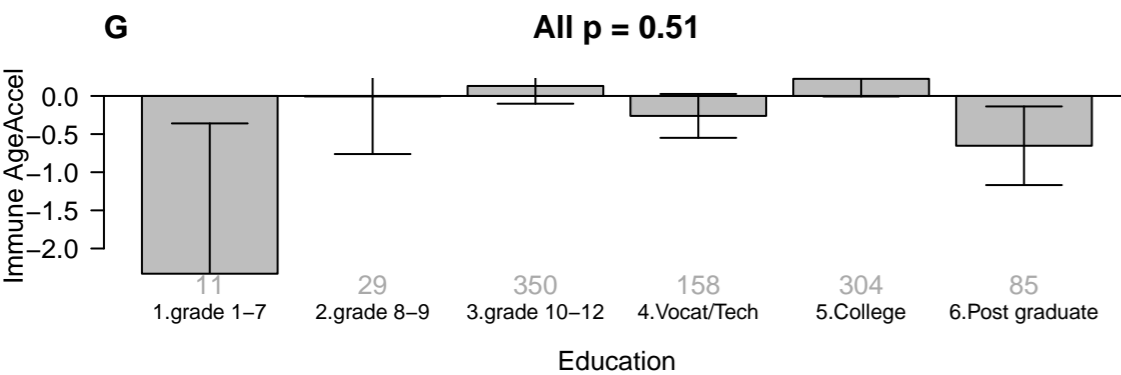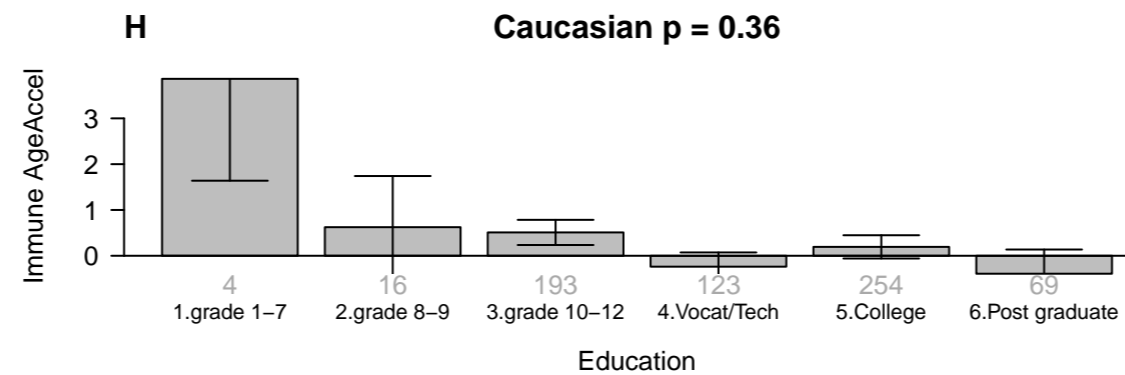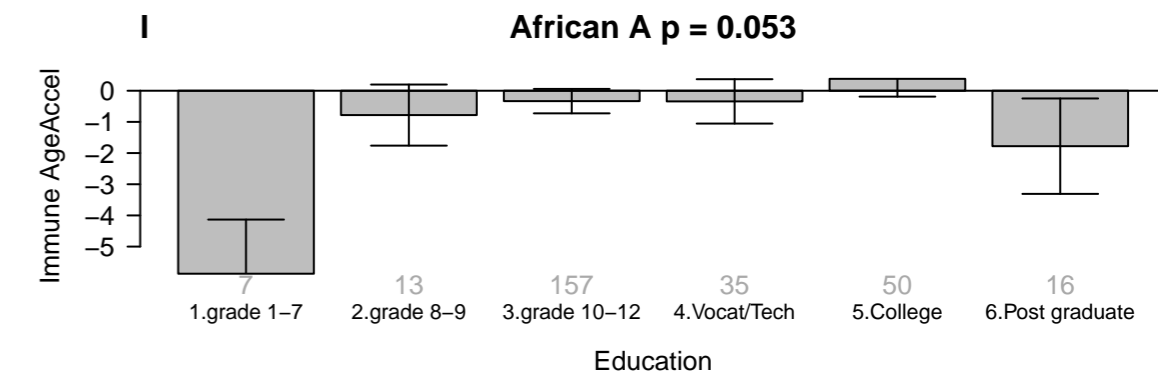

Supplement: Additional file 7: — Educational level versus age acceleration in the Bogalusa study. Each row relates educational level (x-axis) to three respective measures of epigenetic age acceleration: (A-C) Age Accel., (D-F) IEAA, and (G-I) EEAA. The columns correspond to different groups. The first, second, and third columns report findings for (A, D, G) all participants, (B, E, H) Caucasians, (C, F, I) African Americans, respectively. Each bar plot reports the mean values, 1 standard error, and the p value from a non-parametric group comparison test (Kruskal–Wallis). Education was grouped as follows: group 1 = grades 1–7; group 2 = grades 8–9; group 3 = grades 10–12; group 4 = vocational/tech training; group 5 = college; group 6 = postgraduate. (PDF 5 kb) [file 13059_2016_1030_MOESM7_ESM.pdf]

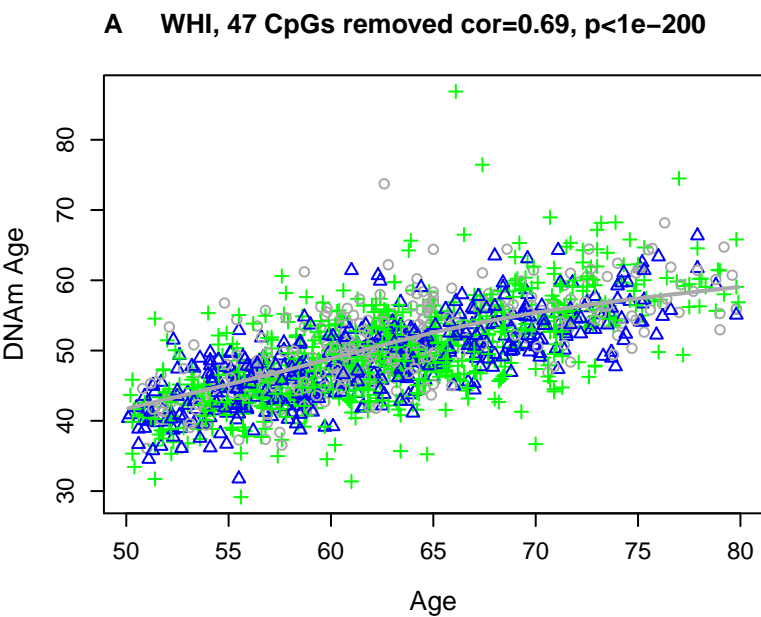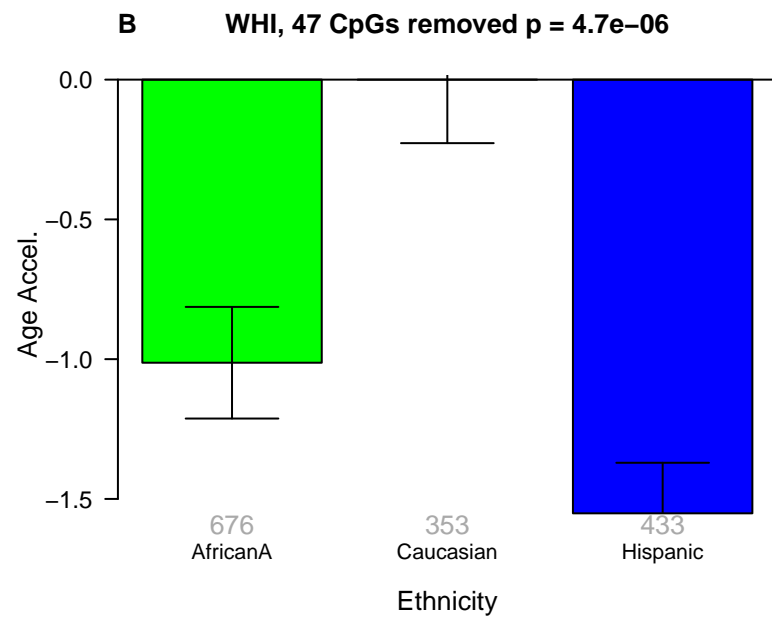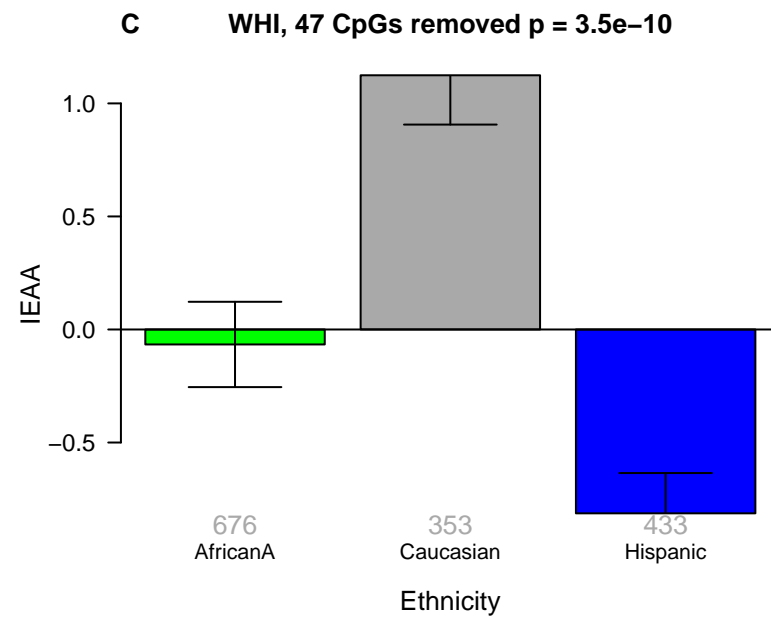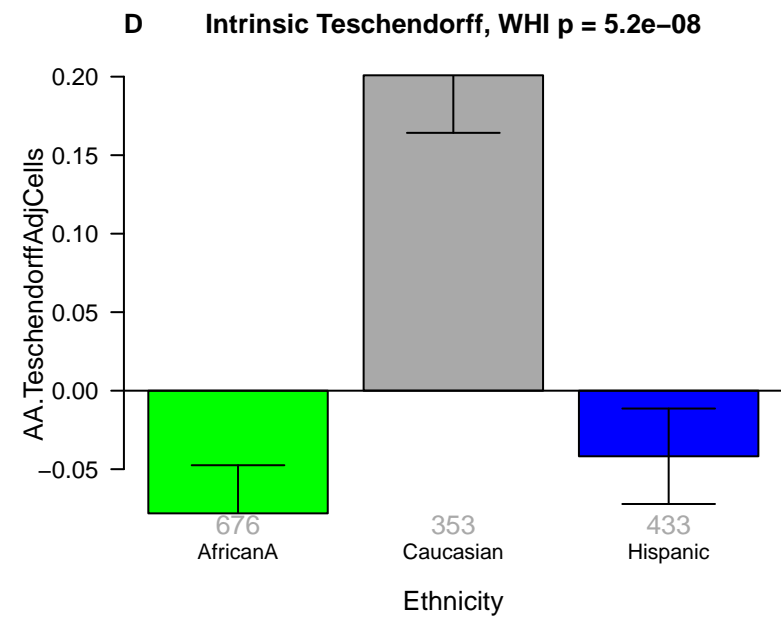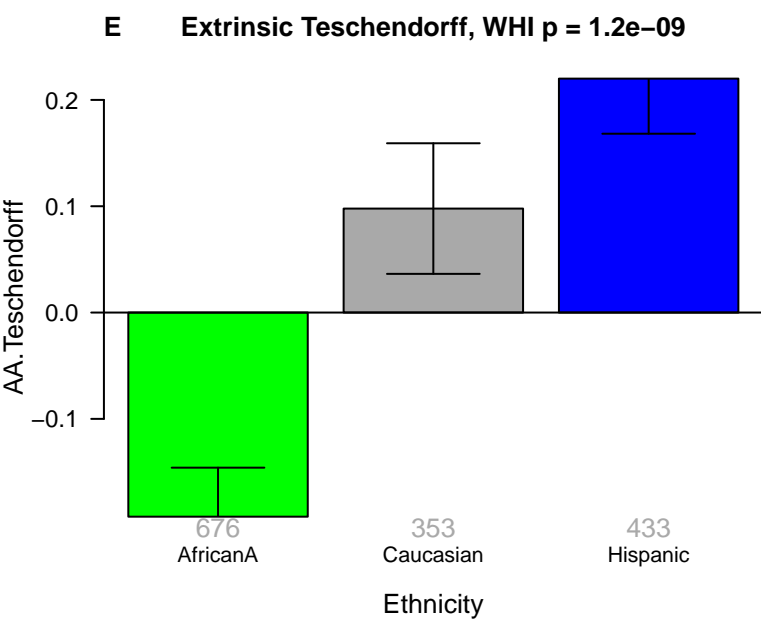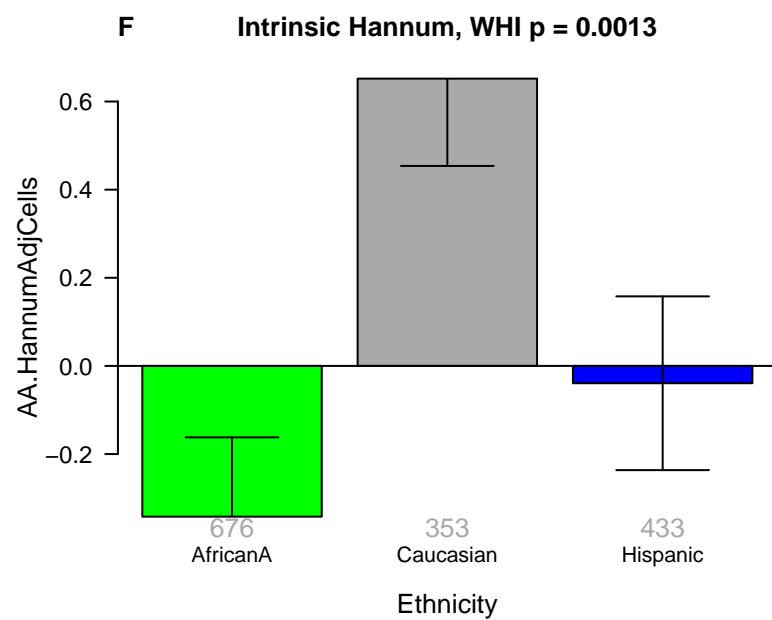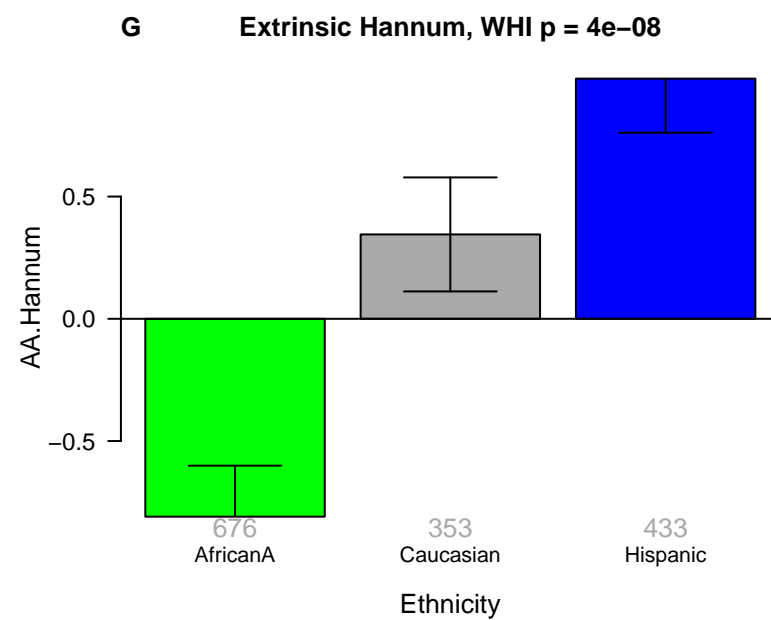

Supplement: Additional file 8: — Robustness analysis with respect to other epigenetic biomarkers of aging in the WHI. A-C Results for the Horvath method when 47 out of 353 CpGs were removed from the epigenetic clock (because they are in the vicinity of a SNP). Since none of the remaining clock CpGs are near a SNP, the resulting age acceleration is not trivially related to race/ethnicity. A DNA methylation age versus chronological age. B Ethnicity versus age acceleration (defined as residual resulting from regressing DNAm age on chronological age). C Intrinsic epigenetic age acceleration versus ethnicity. D, E Alternative epigenetic biomarker of aging based on 589 age-related CpGs from Teschendorff [13]. The biomarker was defined using the following steps. First, the DNA methylation levels of each CpGs were standardized (to mean zero and variance 1). Second, a weighted average was formed by multiplying each CpG by the T test statistic from the chronological age relationship based on the table from the original reference. Third, the weighted average was regressed on chronological age to arrive at a residual. The resulting residual is referred to as extrinsic measure of age acceleration since it was not adjusted for blood cell counts. Fourth, the resulting measure was regressed on estimated blood cell counts (analogous to those used for IEAA) in order to arrive an intrinsic measure of age acceleration. F, G Epigenetic measures of age acceleration using the Hannum method 71 CpGs [19]. D, F Results for intrinsic measures, i.e. measures of age acceleration that adjust both for blood cell counts and chronological age. E, G reports extrinsic measures, i.e. no adjustment for imputed blood cell counts. Each bar plot depicts 1 standard error and reports the results from a Kruskal–Wallis test. (PDF 55 kb) [file 13059_2016_1030_MOESM8_ESM.pdf]
