# Supplementary material for: An epigenetic clock analysis of race/ethnicity, sex, and coronary heart disease
Source: Genome Biol. 2016 Aug 11;17:171. doi: 10.1186/s13059-016-1030-0 (PMC4980791; doi:10.1186/s13059-016-1030-0)
Supplement: Additional file 9: — Demographic and physiologic characteristics of women from the WHI. Case-control status refers to CHD. Two designs were used to select samples: case/control and case-cohort. (DOC 48 kb) [file 13059_2016_1030_MOESM9_ESM.doc]

**Additional file 9. Demographic and physiologic characteristics of women from the WHI. Case control status refers to coronary heart disease. Two designs were used to select samples: case/control and case-cohort.**

| **Variable** | **Case-control set** |  | **Case-cohort sample** |  |
| --- | --- | --- | --- | --- |
|  | **Case (N=637)** | **Control (N=631)** | **Cases (N=432)** | **Sub cohort (N=472)** |
| **Race/ethnicity (Form 2)** |  |  |  |  |
| White | 296 (46.5%) | 292 (46.3%) | 210 (48.6%) | 236 (50.0%) |
| Black | 231 (36.3%) | 229 (36.3%) | 112 (25.9%) | 118 (25.0%) |
| Hispanic | 110 (17.3%) | 110 (17.4%) | 110 (25.5%) | 118 (25.0%) |
| **Age** |  |  |  |  |
| 50-54 | 58 (9.1%) | 58 (9.2%) | 34 (7.9%) | 57 (12.1%) |
| 55-59 | 104 (16.3%) | 104 (16.5%) | 69 (16.0%) | 88 (18.6%) |
| 60-64 | 142 (22.3%) | 140 (22.2%) | 84 (19.4%) | 97 (18.9%) |
| 65-69 | 165 (25.9%) | 163 (25.8%) | 113(26.2%) | 125 (20.6%) |
| 70-74 | 125 (19.6%) | 125 (19.8%) | 85(19.7%) | 68 (14.4%) |
| 75-79 | 43 (6.8%) | 41 (6.5%) | 47 (10.9%) | 37 (7.8%) |
| **Enrollment year** |  |  |  |  |
| 93-94 | 26 (4.1%) | 25 (4.0%) | 32 (7.4%) | 27 (5.7%) |
| 95-96 | 280 (44.0%) | 283 (44.8%) | 191 (44.2%) | 192 (40.7%) |
| 97-98 | 331 (52.0%) | 323 (51.2%) | 209 (48.4%) | 253 (53.6%) |
| **Hysterectomy at baseline** | 333 (52.3%) | 329 (52.1%) | 199 (46.1%) | 187 (39.6%) |
| **Study component** |  |  |  |  |
| HT | 401 (63.0%) | 395 (62.6%) | 277 (64.1%) | 306 (66.3%) |
| Non-HT CT | 92 (14.4%) | 92 (14.6%) | 65 (15.1%) | 73 (14.6%) |
| OS | 144 (22.6%) | 144 (22.8%) | 90 (20.8%) | 93 (19.2%) |
| **Had GWAS data** | 625 (98.1%) | 620 (98.3%) |  |  |
| **Incident event** |  |  |  |  |
| Angina | 66 (10.4%) |  | 81(18.8%) | 4 (12.5%) |
| Angina & CHD | 91 (14.3%) |  | 44 (10.2%) | 5 (15.6%) |
| Angina & Revascularization | 18 (2.8%) |  | 4 (0.9%) | 0 (0%) |
| Revascularization | 125 (19.6%) |  | 83 (19.2%) | 5 (15.6%) |
| Revasc. & CHD | 128 (20.1%) |  | 62 (14.3%) | 3 (9.4%) |
| CHD | 209 (32.8%) |  | 158 (36.6%) | 15 (46.9%) |
